# Quantifying spatial variability in shell midden formation in the Farasan Islands, Saudi Arabia

**DOI:** 10.1371/journal.pone.0217596

**Published:** 2019-06-12

**Authors:** Niklas Hausmann, Matthew Meredith-Williams, Katerina Douka, Robyn H. Inglis, Geoff Bailey

**Affiliations:** 1 Max-Planck-Institute for the Science of Human History, Jena, Germany; 2 University of York, Heslington, United Kingdom; 3 La Trobe University, Bundoora-Melbourne, Australia; 4 Flinders University, Adelaide, Australia; University of Edinburgh, UNITED KINGDOM

## Abstract

During the past decade, over 3000 shell middens or shell matrix deposits have been discovered on the Farasan Islands in the southern Red Sea, dating to the period c. 7,360 to 4,700 years ago. Many of the sites are distributed along a palaeoshoreline which is now 2–3 m above present sea level. Others form clusters with some sites on the shoreline and others located inland over distances of c. 30 m to 1 km. We refer to these inland sites as ‘post-shore’ sites. Following Meehan, who observed a similar spatial separation in shell deposition in her ethnographic study of Anbarra shellgathering in the Northern Territory of Australia, we hypothesise that the shoreline sites are specialised sites for the processing or immediate consumption of shell food, and the post-shore sites are habitation sites used for a variety of activities. We test this proposition through a systematic analysis of 55 radiocarbon dates and measurement of shell quantities from the excavation of 15 shell matrix sites in a variety of locations including shoreline and post-shore sites. Our results demonstrate large differences in rates of shell accumulation between these two types of sites and selective removal of shoreline sites by changes in sea level. We also discuss the wider implications for understanding the differential preservation and visibility of shell-matrix deposits in coastal settings in other parts of the world extending back into the later Pleistocene in association with periods of lowersea level. Our results highlight the importance of taphonomic factors of post-depositional degradation and destruction, rates of shell accumulation, the influence on site location of factors other than shell food supply, and the relative distance of deposits from their nearest palaeoshorelines as key variables in the interpretation of shell quantities. Failure to take these variables into account when investigating shells and shell-matrix deposits in late Pleistocene and early Holocene contexts is likely to compromise interpretations of the role and significance of shell food in human evolutionary and socio-cultural development.

## Introduction

Globally, shell middens, that is sites in which discarded mollusc shells are the dominant physical constituent of the deposit, also known as shell-matrix deposits, are typical coastal sites [1–7]. They serve as important archives of past human life, as they contain not only abundant remains of mollusc shell but also provide a chemical and structural environment that protects other archaeological and biological remains [8–10]. Because of this, shell midden sites provide archaeological information on prehistoric and pre-contact hunter-fisher-gatherer and agricultural societies in many coastal landscapes of the world, as well as ecological information about their associated climate and environment [11–14]. Shell-matrix sites composed of freshwater mollusc-shells or terrestrial molluscs are also known in inland locations in many parts of the world[15,16], however in this paper we focus on coastal middens and marine molluscs in a mobile hunter-gatherer context. The large quantities of shells resulting from shell food consumption, their relative durability and resistance to decay, and their tendency to form substantial mounds of high archaeological visibility have all encouraged a long history and variety of studies devoted to such issues as chronology, site function, palaeodiet, palaeoeconomy, and the long-term history of coastal adaptations and their evolutionary consequences, with an extensive literature on the appropriateness and accuracy of quantitative measures such as the volume of shell-matrix deposits, numbers of shells collected, shell-to-meat-weight ratios, and comparisons with other food remains [4,17–23]. This is true both for mid to late Holocene periods, which are well known for the ubiquity of their shell matrix sites, as well as for early Holocene and late Pleistocene periods, where coastal evidence is much more elusive, as sea level change would have submerged most coastlines for much of this period [24–26].

A major obstacle in these studies is the fact that mollusc shells are much more resistant to decay and destruction than other food remains and are therefore liable to over-representation and to exaggerating the relative importance of shell gathering activities and shell food in past subsistence. A 10-metre-thick shell mound with 10,000 cubic metres of shell sounds like a vast abundance of food, and a physically prominent landscape feature. However, if radiometric dates, radiocarbon or otherwise [27], show that the mound took several hundred years to accumulate, it can be demonstrated that this volume of shells, if spread evenly throughout the duration of the mound, would have represented much less food than suggested by first impressions[28]. With the advent of radiocarbon dating, especially the availability of funding for multiple sequences of radiocarbon dates in recent years, and the opportunities this creates for statistical modelling of large samples and high-resolution dating, attention has switched to measures of inter-site and intra-site rates of accumulation as a more useful source of information, opening up a range of new questions and interpretations, including new insights into site formation processes [23,29–35]

Nevertheless, comparisons of rates of accumulation are subject to two potential biases. Both refer to taphonomic effects, i.e., the variable processes that determine the deposition, exposure, burial, preservation and visibility of material remains, and these may operate differently on shell deposits of different types in different contexts [32,33]. One is the impact of in situ decay processes on shell accumulations. The other is the differential impact of post-depositional processes acting on shell accumulations in different landscape settings and the selective destruction of sites by sea-level change or erosion at the shore edge in comparison with those located further away from the shoreline. We consider both biases in this paper, though we place particular emphasis on the latter in the Farasan context.

Regarding in situ effects, recent work has highlighted the dual-character of shells as food refuse and as carbonate sediment, which naturally undergoes post-depositional pedological processes, revealing diagenetic processes that influence the present thickness (and therefore volume) of shell-bearing layers, [9,33,36–39]. Diagenetic processes also include trampling and exposure to weathering, which differentially affect shells of different robustness and shell deposits of different ages and degrees of exposure. Other less obvious factors are carbonate dissolution, increased compaction in deeper layers, and the development of micro-environments that change the preservation conditions within layers and therefore the preservation of the shells within them.

Regarding the second taphonomic variable, it is a well-established fact, inherent in the labour costs of transporting molluscs, that the high ratio of shell-weight to meat-weight for most marine molluscs promotes the processing of shell food as close as possible to the source of supply, that is on or very close to the immediate shoreline [40–48]. Only some of the unprocessed shell may be carried further inland, over distances of hundreds of metres to kilometres, to sites that are located more conveniently for other reasons–shelter, better access to freshwater and other food supplies or other factors. But the quantities of unprocessed shell carried over these greater distances are likely to be relatively small, with a sharp fall-off of quantities with increasing distance. Other variables may override this distance constraint, for example large molluscs with higher meat-to-shell weight ratios, use of boats or pack animals for transporting larger quantities over greater distances, use of shells for purposes other than food [49,50], collection of shells for ceremonial feasting [40], or as building material [38]. But we treat these as exceptions to the general rule and they are mostly not relevant to the Farasan context. Also, the actual meat may be preserved after removal of the shells and carried elsewhere for consumption or even carried over longer distances as part of extensive trade networks [47], but the material by products of shell processing will in general conform to the above distance constraints.

The result is likely to be a site distribution like that described by Meehan [40] with home bases situated away from the shore with relatively small quantities of shells, and processing sites located at the shoreline containing the bulk of shell material, or sites used for immediate consumption of molluscs (dinner-time camps in Meehan’s classification) also located on the shoreline or as close as possible to it. Of course, sites conveniently located for shell processing may also be habitation sites if the location is suitable for other activities, and site location by itself is not necessarily a good guide to the function of individual shell middens.

The main consequence of these distance constraints from a taphonomic point of view is that sites located on the immediate shoreline are likely to be far more vulnerable to damage, destruction or burial under marine sediment because of changes in sea-level, or other geomorphological processes acting on the shoreline such as erosion or accumulation of sediment, in contrast with sites situated even a short distance further inland [39,51–54]. This is especially likely for large shell-matrix deposits associated with processing of molluscs available in large quantities on shallow intertidal substrates. These locations are often associated with shallow offshore topography and low shorelines of limited relief. They are therefore especially sensitive to small rises in sea-level, which can easily destroy shell deposits by wave action or bury them under marine sediment, or to lateral erosion or sedimentation even when sea-level is stable. The result may be a site distribution that is missing a whole class of shell-matrix deposits, and therefore unrepresentative of the original pattern, and this is especially likely when looking at site distributions created during periods when sea level was lower than the present. Conversely, home-base sites situated inland from the shore zone are likely to be better protected from sea-level rise but more vulnerable to disturbance and damage by site-cleaning activities and prolonged exposure to weathering and foot traffic, in contrast to processing sites on the shore where rapid accumulation of shells and consolidation of thick deposits provides better protection from damage by physical weathering and in situ human activities.

Our aim in this paper is to examine these inter-site variations in rates of shell accumulation using the shell middens of the Farasan Islands as our case study and to consider the wider implications of our results in terms of shell food quantification, site function and taphonomic effects. The Farasan Islands are particularly well suited to this aim. Over 3,000 open-air shell middens have been recorded on the Islands, providing the necessary data to study spatio-temporal distribution patterns. Sites range in size and character from large mounded deposits on the shore to small shell deposits or scatters at variable but relatively short distances (hundreds of metres) inland. Furthermore, sites date to the period between c. 7,360 and 4,700 years ago, during which Holocene sea-level in the region rose to a peak during the mid-Holocene Highstand at 6,000 years ago before subsequently falling to near-modern levels [55]. This provides an appropriate context for assessing differential taphonomic effects associated with sea-level change. Moreover, because of the remoteness of the Islands, low precipitation, the small resident population and the lack of tourism until recently, there has been relatively little damage or destruction of sites by modern road-building or other construction activities. The result is a distribution of shell middens that is probably as close to a pristine distribution as it is possible to find. Also, the Farasan middens form generally discrete deposits that do not overlap and have relatively deep stratigraphies that facilitate dating and inter-site comparison. These factors enhance their suitability for the investigation of differential rates of accumulation in different types of locational settings.

## Palaeogeographical and archaeological context

**Fig 1:**
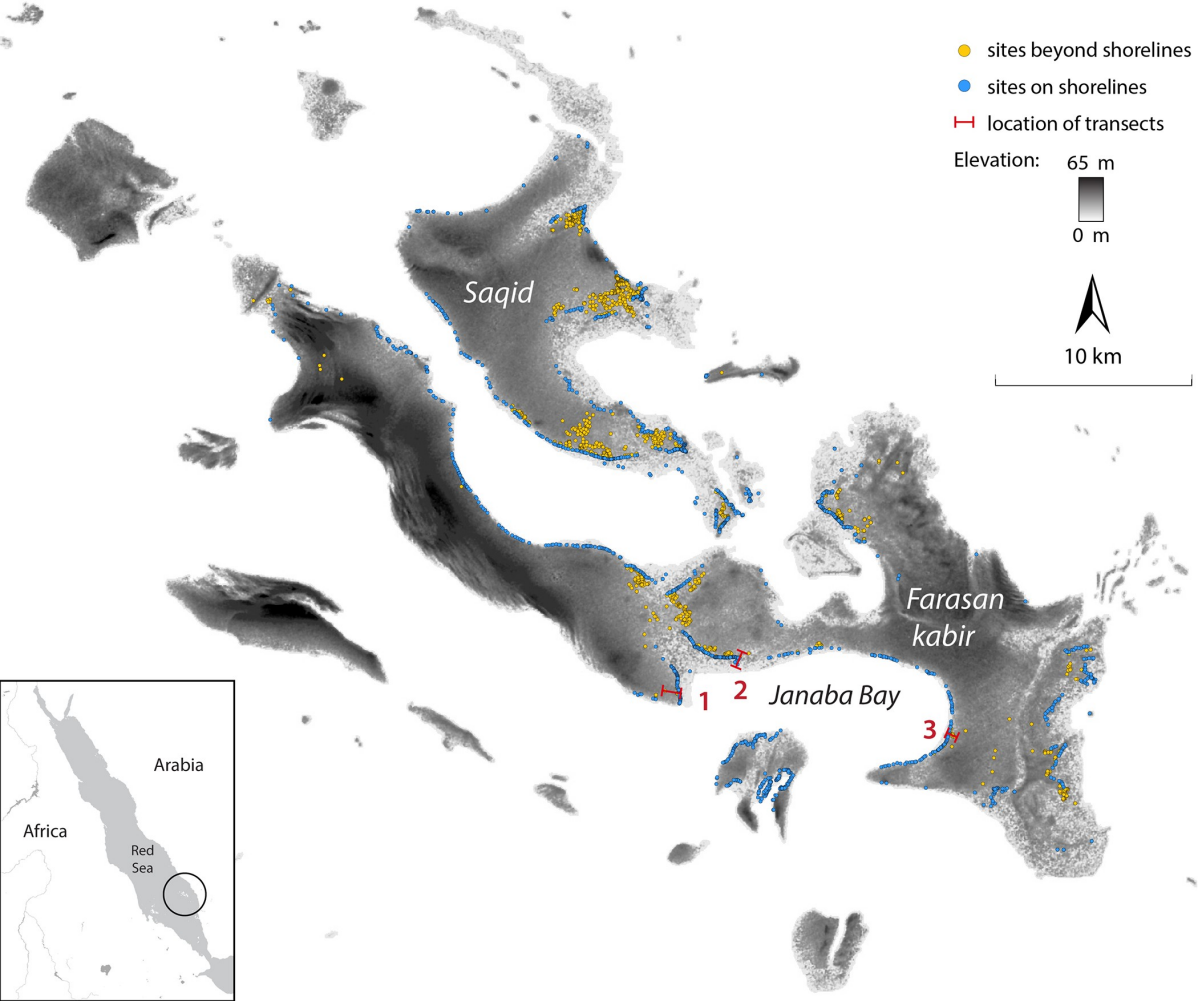
Elevation map of the Farasan Islands, showing the spatial distribution of shell midden sites. Blue dots are shoreline middens, yellow dots are post-shore sites. The three transects with the sites selected for detailed radiocarbon dating are all in Janaba Bay and are shown in red. We refer to these as Janaba West (1), Janaba West-Central (2), and Janaba East (3).

The Farasan Islands are located in a fertile marine environment in the southern Red Sea between East Africa and the Arabian Peninsula, a crucial junction for population movements and cultural exchange between the two landmasses [56–59]. They comprise over 120 islands of varying size, the two largest being Farasan Kabir and Saqid (Fig 1).

**Fig 2:**
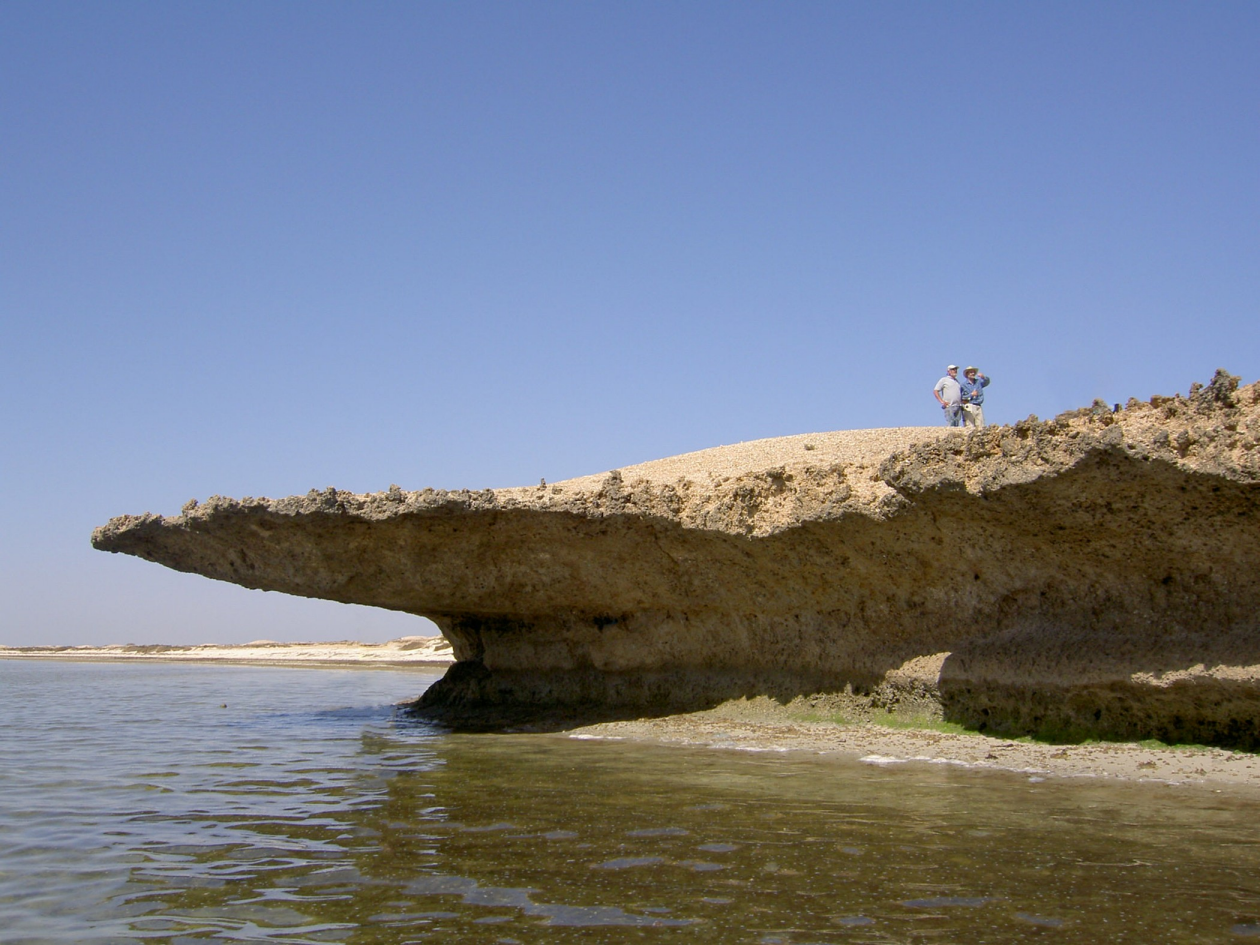
The edge of the coral platform in Janaba East, showing a notch deeply undercut by marine erosion. Above it is a shell mound located with two figures standing on it. This undercut terrace is of variable extent along the Janaba Bay shoreline and is not present on the shoreline in the background nor associated with the sites in Transects 2 and 3, where the shallow gradient onshore and offshore has resulted in an almost imperceptible break between the landward surface of the fossilised coral platform and the offshore surface, Photo by Garry Momber, May 2006.

They are composed of fossilized coral reefs and limestone uplifted by the mobility of underlying Miocene evaporites (salt deposits) and are now some 40 km offshore of the Saudi Arabian mainland. Sea-level rise following the Last Glacial Maximum flooded extensive areas of continental shelf in the southern Red Sea and isolated the Farasan Islands from the Arabian mainland [57,59–61]. In the southern Red Sea relative sea-level reached the modern position at about 7,000 cal BP, and then rose by a further ~2 m to reach a mid-Holocene highstand (henceforth ‘Highstand’) at ~6,000 cal BP, after which it dropped back again to the present level [55,62]. The present-day land surface is mainly a coral platform of low relief with patches of thin soil, limited vegetation cover, an annual rainfall of 50–100 mm, no permanent freshwater bodies, a native mammalian fauna of gazelle (*Gazella gazella farsani*) and a highly productive marine environment with abundance of fish and shellfish [63–66]. On many shorelines the coral platform has been eroded by marine action at the shore edge to create a low cliff typically ~2–3 m high with a characteristic undercut notch (Fig 2). The height of this cliff varies in different parts of the Islands because of localized tectonic warping associated with ongoing salt tectonics, reaching as high as 5 m in some areas and reducing almost to nothing on other shorelines. These localised differences appear to be a function of the onshore topography (steeper or shallower as the case may be) and localised tectonic uplift, some of which is recent and postdates the accumulation of the shell mounds. Many of the shell middens including some of the mounds are located on the edge of the coral platform above an undercut notch, but many more are located on shorelines with a shallow topography where this feature is absent. This is especially the case around the inner edges of shallow bays that once formed intertidal sandflats with extensive beds of marine molluscs, and it is around these shallow bays that the largest concentrations of shell middens are found. These bays have since dried out because of a combination of infilling by sand, minor marine regression after the Highstand and tectonic uplift [62].

Because the landscape is quite arid with little vegetation cover, the shell middens are distinctive physical features of the coastal landscape. With their whitish colour they stand out against the darker background of the land surface and are visible over long distances and on satellite images. This applies even to small surface scatters as well as the bigger mounds. Similar sites have been identified from satellite imagery on the Dahlak islands on the Eritrean side of the Red Sea opposite the Farasan Islands [60,62]. The shell middens vary in size from shell scatters 5–10 m in diameter to conical mounds up to 5 m high and 30 m long. Most deposits are circular or oval in plan, though scatters can be more irregular, and the largest scatters cover an area of hundreds of square metres. The majority of deposits lie somewhere between these extremes, and we define as mounds sites with at least 1 m thickness of deposits, although we also note that this is an arbitrary threshold, and that site size and thickness form a continuum across this boundary. Also, it is not always possible to identify the thickness of what appear from surface observations to be scatters or thin deposits without excavation; those that we have excavated usually have shell deposits of no more than c. 50 cm thickness. Raw materials for making stone artefacts are scarce on the Islands, and relatively few artefacts have been found in the middens. These include occasional potsherds, small manuports of coral and limestone and flakes made from *Tridacna* shell. Stone tools and potsherds are more prevalent on surface scatters than in deeply stratified mounds, suggesting differences in site function which we comment on later. In deeply stratified mounds, the shell deposits are interleaved with ash lenses, and fish bones and occasional bones of gazelle are also present. The midden deposits contain a wide range of edible marine molluscan species of reef and sandflat habitats, with the dominant species comprising the small gastropod *Conomurex fasciatus*. One mound contains two human burials [65]. We give further details of site composition later for those deposits selected for detailed analysis in this paper.

Shell middens occur in two types of locations. Some, including the largest shell mounds are located on the palaeoshoreline at the edge of the coral platform (n = 1,728), and we refer to these as ‘shoreline’ sites. Others are found farther inland on the land surface at distances that range from ~30 m to 1 km or more from the shoreline (n = 1,282). In other regions or contexts, the latter sites would be called ‘inland sites’, but because the Farasan Islands are 40 km off the coast of the Arabian mainland and the shoreline is never further than 5 km from any point on Farasan, we refrain from using the term ‘inland sites’ and instead uses the term ‘post-shore’ sites. These post-shore sites are generally low mounds or shell scatters. More importantly, in the context of coastal erosion, the post-shore sites are at a distance from the shoreline and at an elevation above it that protects them from the destructive impact of wave-action and sea-level rise. This variation in site locations results in two characteristic types of site distribution: linear patterns that closely follow the palaeoshorelines and clustered distributions–concentrations of sites that include mostly post-shore sites and also some shoreline sites bordering the adjacent palaeoshoreline (Fig 1).

We hypothesise that the post-shore middens have a different function from the sites located directly on the shoreline, and correspond to habitational sites in Meehan’s [40] classification, whereas the sites situated directly on the shore could correspond to a variety of functions including dinner-time camps–sites used expediently for the consumption of molluscs at the time of collection–and processing sites in Meehan’s terminology, as well as habitation sites. However, we do not prejudge the issue of differential site function from site location alone. Our main interest is in the relationship between rates of shell discard and site location. In this regard, we hypothesise that the highest rates of shell accumulation are in the shoreline middens because it is here where we expect that the bulk of shell processing would have been carried out, and that rates of shell accumulation in post-shore sites should be consistently lower because of their greater distance from the shoreline. We further hypothesise that the earliest dated middens are in the post-shore group because these are the ones most likely to have survived from the period before sea-level rose to reach the Highstand. We hypothesise that during the period of sea-level rise prior to the Highstand, a significant number of shell middens could have existed, only to have been destroyed or lost to view by the subsequent sea-level rise. This would skew our interpretation of the shell midden cluster as a whole by removing early-dated processing sites located directly on the shore, which would contain the bulk of discarded shells, while leaving intact the post-shore sites of the same period.

## Materials and methods

### Site selection

**Fig 3:**
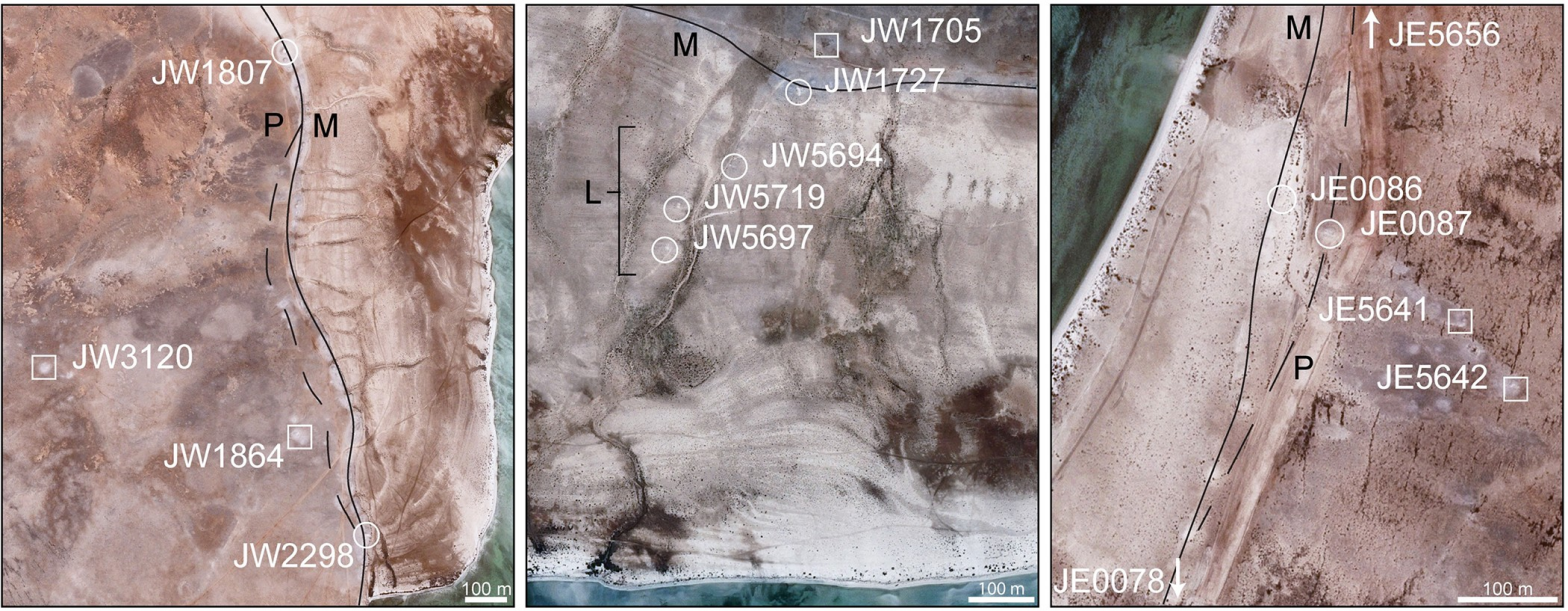
Sample transects showing the locations of radiocarbon-dated shell-midden sites superimposed on satellite imagery. Shoreline sites are indicated with a circle, post-shore sites are indicated with squares. Shorelines are indicated as follows: M: main shoreline, continuous black line; P: peak shoreline, dashed black line; L: lower shorelines, of which there are three in Transect 2, roughly parallel with the main shoreline as indicated by the square bracket.

**Fig 4:**
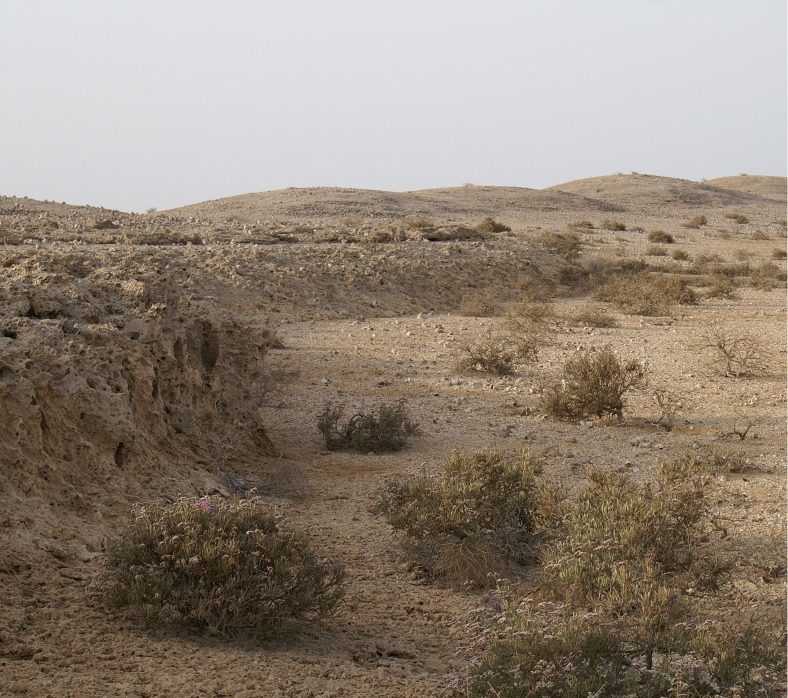
Shell mounds on the west side of the large inlet in Janaba West (Transect 1). Photograph taken facing North-West, the low cliff at the edge of the coral platform on which the mounds sit is clearly visible on the left and represents the palaeoshoreline contemporaneous with the accumulation of the shell mounds. It is not nearly as deeply undercut as elsewhere because of erosion and partial collapse of the original overhang. It is probably the result of localised tectonic uplift that postdates the shell mounds and is not present on the palaeoshorelines elsewhere around this bay, for example in the Janaba central cluster. In the foreground are sandy deposits which extend out from the palaeoshoreline into what was originally a marine inlet. Photo by Geoff Bailey, March 2008.

We selected 15 sites for excavation and collection of dating samples. These sites form transects which encompass the different types of locations that were used for shellfish deposition (Fig 3). All three transects include shoreline and post-shore sites. There are also differences between them that highlight distinctive variations along the Farasan coastline. Transect 1 (Janaba West) is on the west shoreline of what was once a huge shallow marine inlet. It includes some of the largest shell mounds of the region, forming an almost continuous series of mounds along the edge of the palaeoshoreline (Fig 4). The inlet has now dried out and is a flat sand-filled embayment. Transect 2 (Janaba Central) is at the head of this same inlet. It is one of the palaeoshorelines that is now furthest inland from the present shoreline and highlights the retreat of sea level after the Highstand through a series of shorelines that are spatially separated because of the shallow gradient. Transect 3 (Janaba East) includes mounds of varying size on two closely adjacent beach ridges, the innermost of which represents the maximum sea level of the Highstand, and a variety of post-shore sites. Given these features, we further subdivide the shoreline sites into three categories–‘main’, ‘peak’ and ‘low’ (Fig 3). ‘Main sites’ are located on the edge of the coastal platform immediately adjacent to the marine zone from which the live shells were collected. ‘Peak’ sites are situated on a beach ridge forming a palaeoshoreline slightly inland of the main shoreline and formed at the peak of the Highstand. These sites have only been sampled in Transect 3 but also occur close to Transect 1. Here the palaeoshorelines are marked by beach ridges of sand and compacted shell fragments fronting a former shallow bay that is now covered with wind-blown sand. ‘Low’ sites are located on beach ridges that represent shorelines formed when sea-level was retreating from the Highstand and these are only present in Transect 2. ‘Post-shore’ sites are located on the coralline land surface at varying distances inland from the nearest shoreline.

**Fig 5:**
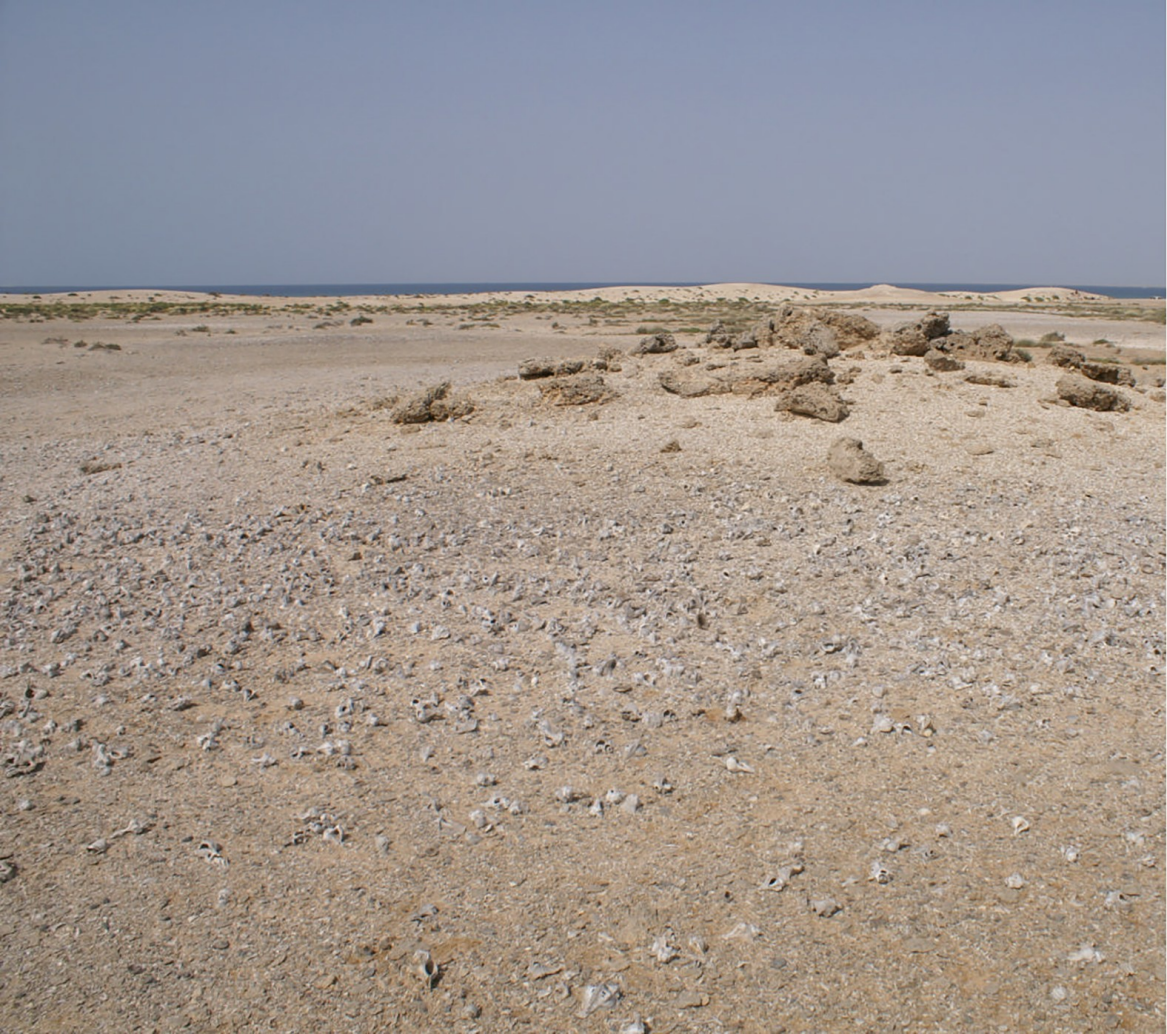
Post-shore shell scatter in the Janaba East cluster associated with Transect 3. The site appears to be a low mound with a deflated shell scatter on the surface and coral blocks representing the remains of structures but has not been excavated or dated. Photograph taken facing West, shell mounds along palaeoshorelines are clearly visible in the distance. To the right is the shell scatter of JE5641, identifiable by the patch of red material, which is the spoil heap from the trench excavated into this deposit. Photo by Geoff Bailey, February 2013.

**Fig 6:**
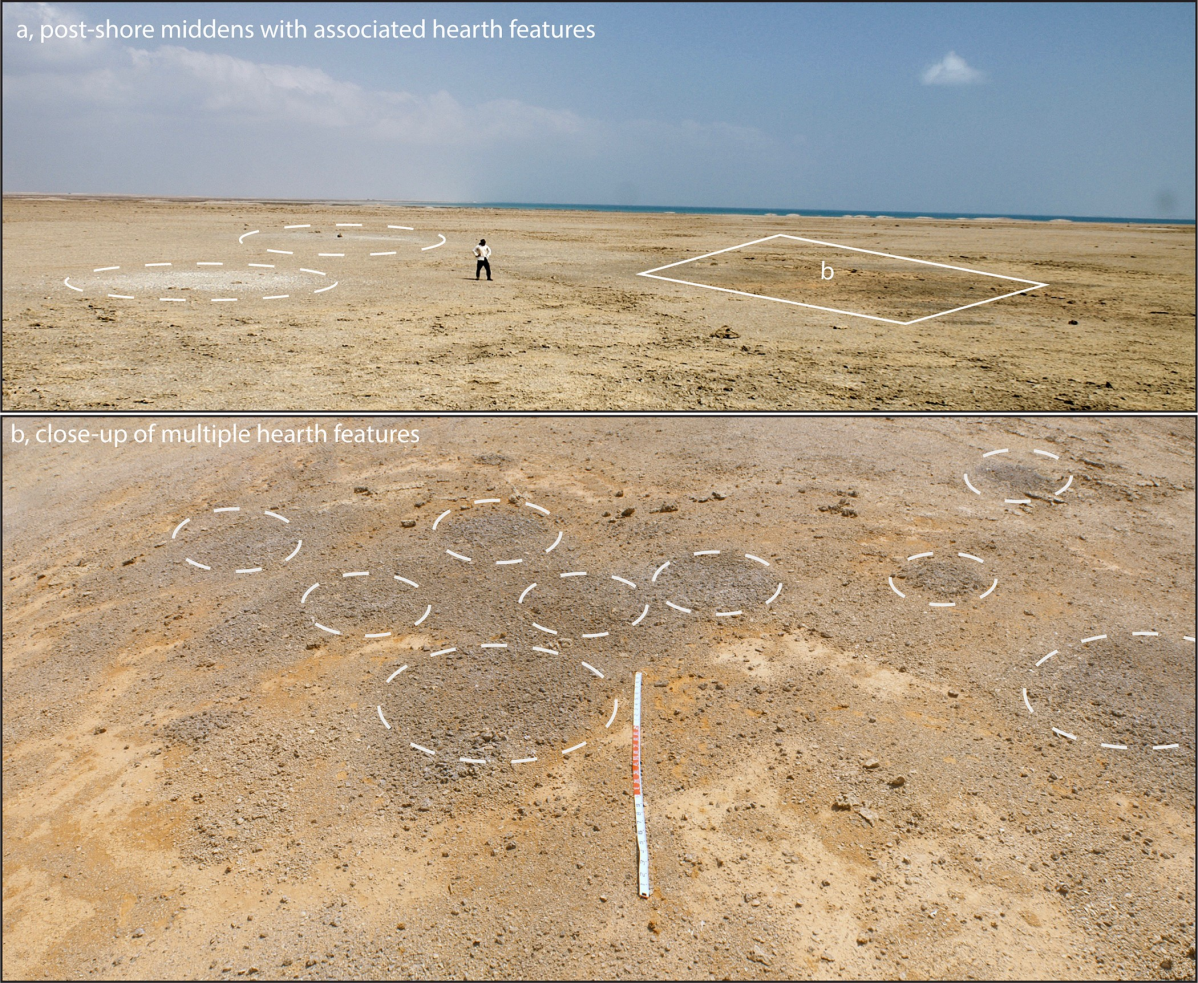
Post-shore scatters. (a) Shallow post-shore shell scatters found close to circular patterns of burnt material in the Janaba East cluster with shell mounds on the main shoreline visible in the distance; b: close up of multiple burnt areas–scale is 3 m. Photo by Niklas Hausmann, February 2013.

**Fig 7:**
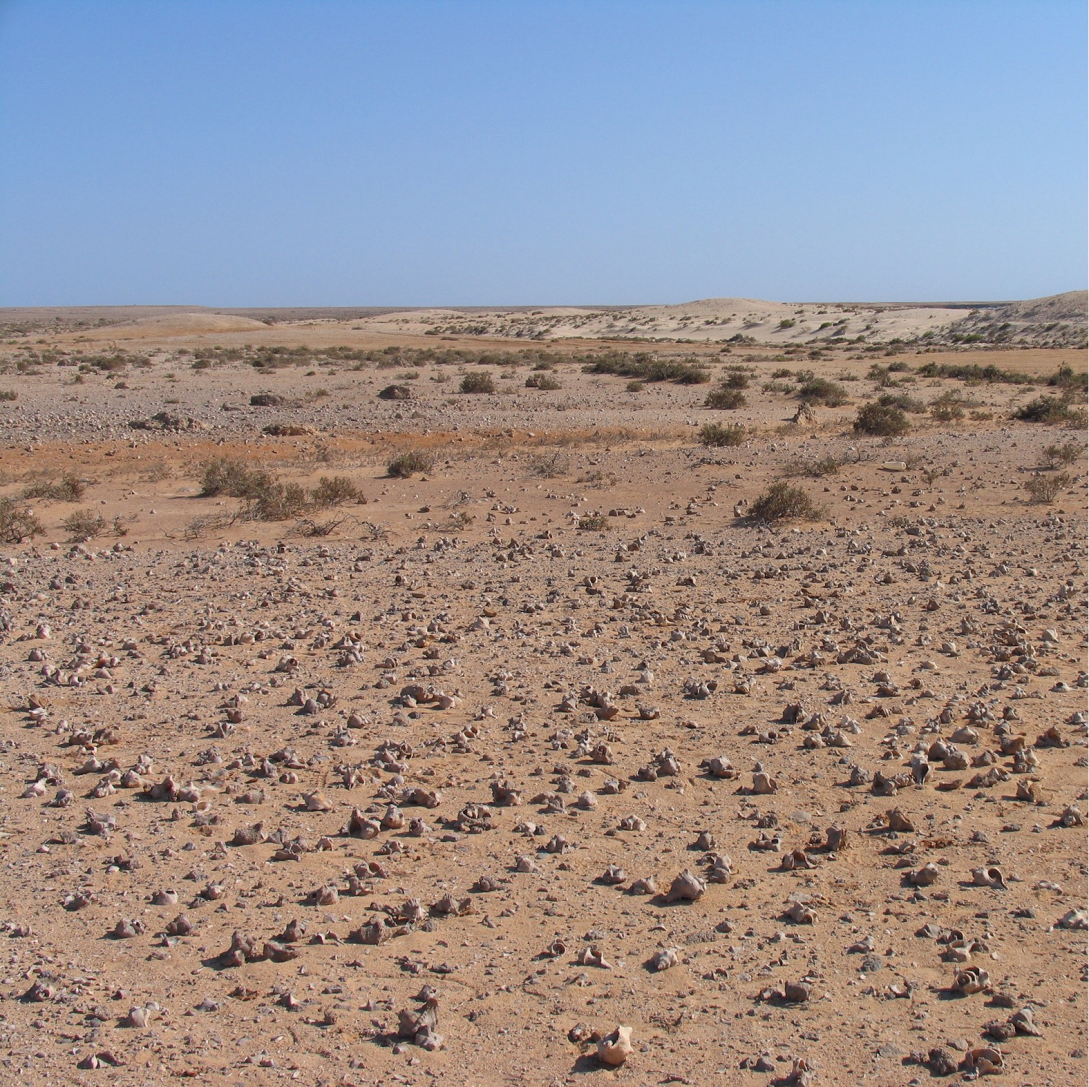
Deflated post-shore site in foreground comprised mainly of shells of the large mollusc, *Chicoreus ramosus*. A second shell scatter is visible in the left middle distance (JE5641). Photograph taken facing South, the large shell mound JE0086 on the main shore is visible in the distance to the right (with a trigonometrical pillar on its summit). A row of shell mounds on the main shoreline extends to the left of JE0086 (see also Fig 6) and a row of peak shoreline sites (incl. JE0087) is found to the far left of the image (see also Fig 3). Photo by Geoff Bailey, May 2006.

Post-shore midden sites are grouped as irregular clusters of individual heaps or scatters; the focal feature for their location is not apparent and may not have been preserved. Remains of structures built of blocks of coral are present in some cases and may have formed these focal points (Fig 5). Closer proximity to other, terrestrial, food sources may be another factor, as seasonality data suggests that shellfish exploitation varied in intensity throughout the year and that other resources took their place most likely including plant foods and gazelle [67]. In addition, many post-shore sites are associated with circular patterns of burned coral bedrock, which were tentatively interpreted as traces of hearths, as they were exclusively found next to middens (Fig 6) and occasionally contained lithic material and burnt shell [66]. Some of the post-shore sites also have quite high numbers of potsherds on their surface, whereas potsherds are present but very rare within stratified layers in the shoreline mounds, occurring there as isolated specimens. There is little information on how permanent the occupation in these post-shore locations was, but the partial overlap of some burned features suggests that they were reused over time. Generally, post-shore shell middens are shallow in profile with a stratigraphy of <1 m. Their main components are shells of *Conomurex fasciatus* occasionally mixed with shells of other mollusc species (*Anadara antiquata*, *Chicoreus ramosus*, *Barbatia* sp.) and occasional mammalian remains (mostly *Gazella* sp.). The majority of post-shore sites are deflated, and a significant number are only matrix-supported scatters with little or no stratigraphic integrity (Fig 7).

**Fig 8:**
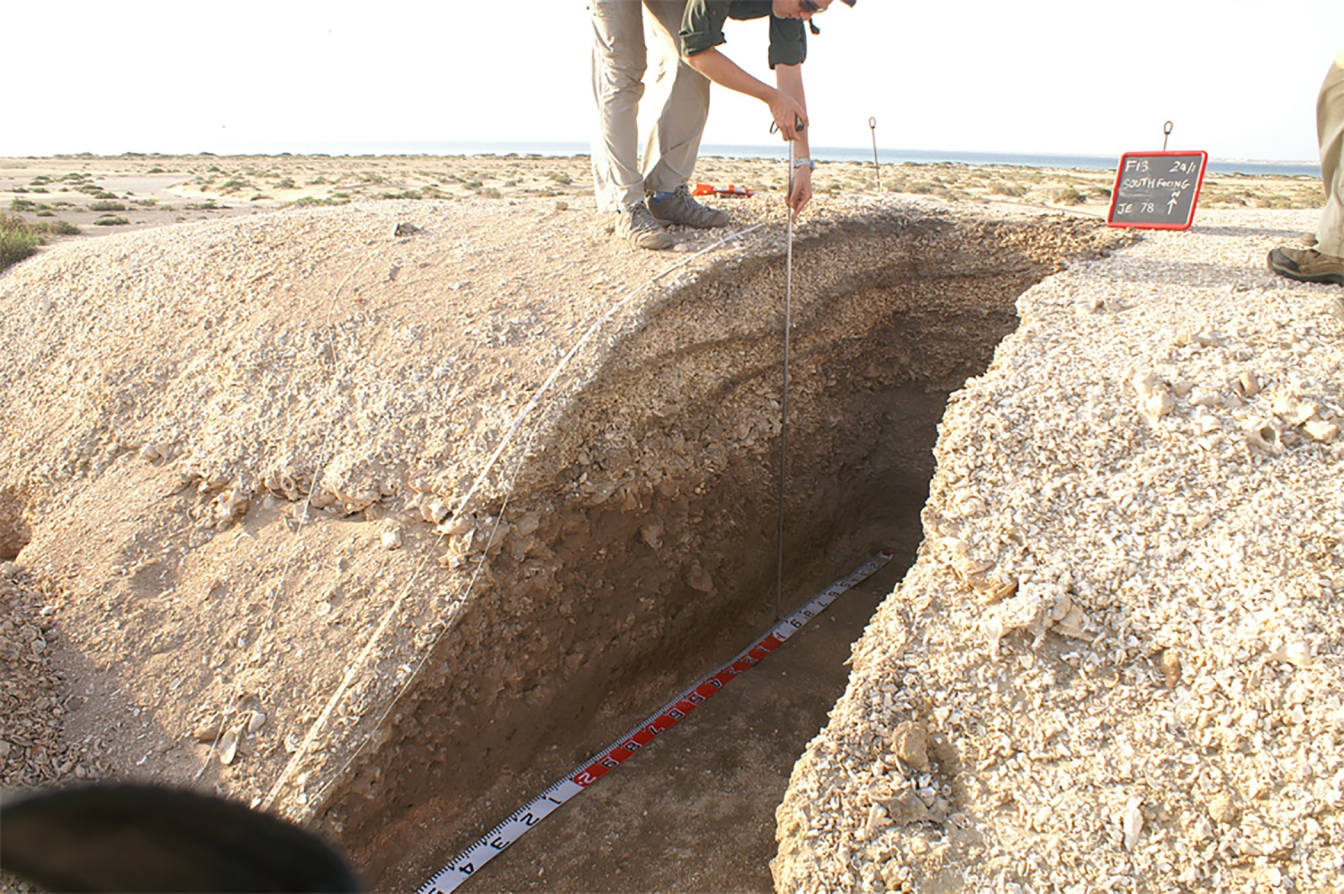
Mound JE0078 on the main shoreline of the Janaba East cluster (Transect 3) after excavation, showing ash lenses clearly visible in section. The white shell matrix in the upper deposit is dominated by shells of *C*. *fasciatus*, the darker shell matrix in the lower layers has a higher proportion of the large gastropod, *Chicoreus ramosus*. Photo by Geoff Bailey, January 2013.

Middens located on the main or peak shorelines are almost entirely more than 1 m in thickness and are clearly mounds in our terminology (Fig 4 and Fig 8). Most mounds have a height of >2 m which, combined with an average diameter of around 25 m, results in an average volume of shell content of well over 200 m^3^. Mounds are well stratified, with distinct layers of ash and charcoal interleaved with clast-supported layers of pure *C*. *fasciatus* or *C*. *fasciatus* mixed with other species (*A*. *antiquata*, *C*. *ramosus*, *Barbatia* sp.), and occasionally single-species-layers of *A*. *antiquata*, *C*. *ramosus*, or *Barbatia* sp. (Fig 8).The mounds generally indicate single purpose activities related to processing freshly caught molluscs on a large scale, although some middens contain fish bones often concentrated in well-defined layers [64].

**Fig 9:**
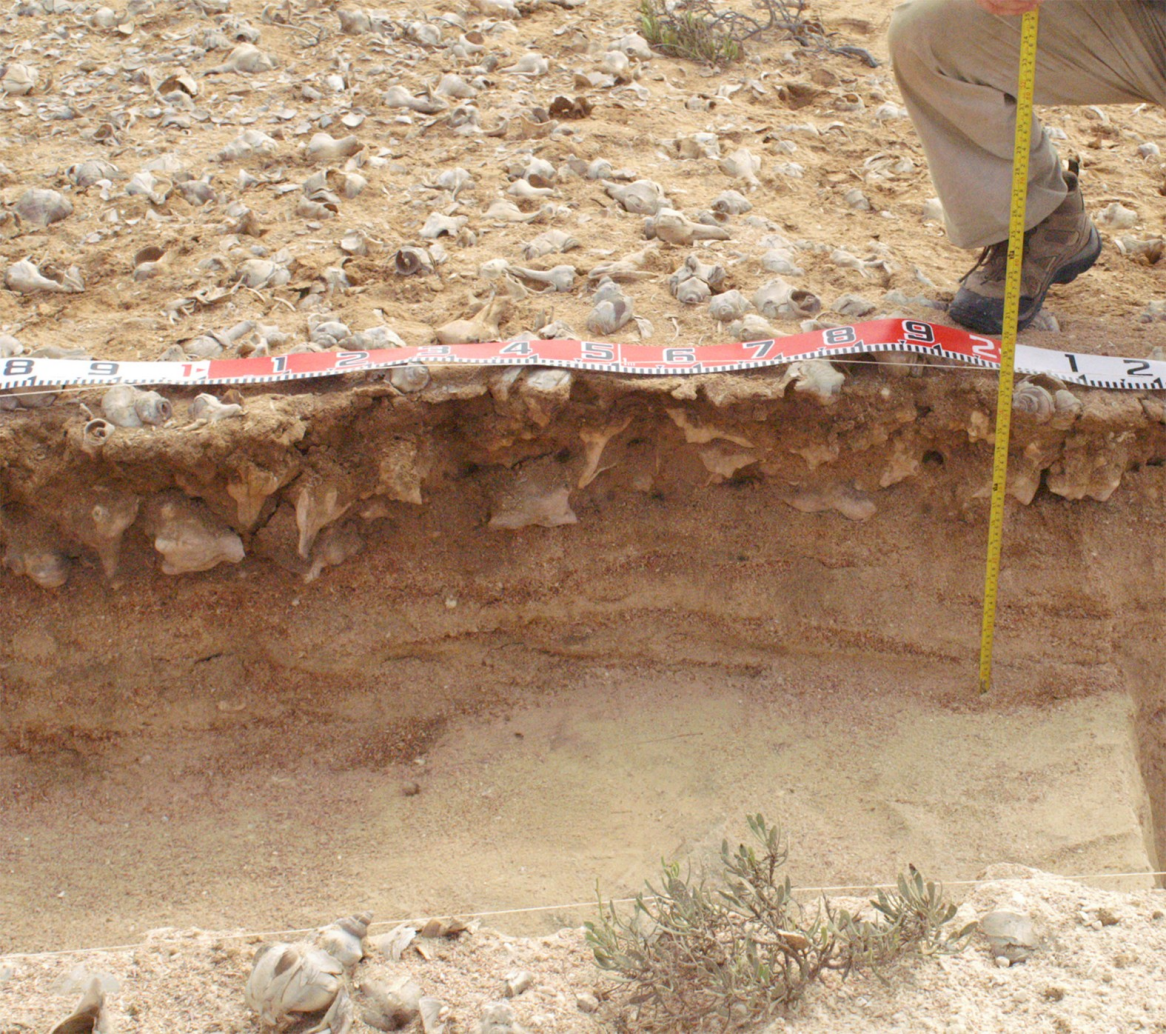
Site JW5697, one of the low-shore middens in the Janaba West-Central cluster on transect 2. The shell matrix is dominated by shells of *Chicoreus ramosus* and sits directly on a thick beach deposit. Photo by Niklas Hausmann, February 2013.

Lastly, sites located on the lower shorelines in Transect 2 (Fig 3), are spread over several shorelines that were likely short-lived. They are thin shell deposits no more than 40 cm deep that have undergone some degree of deflation or disturbance because of the unstable sandy surface beneath them (Fig 9). All are located on low sandy beach ridges with shell scatters extending along the line of the ridge in each case. These ridges represent a succession of short-lived shorelines that tracked the retreat of sea-level after the Highstand. Midden compositions were different from other sites and included vertebrate bones and burnt ceramics, suggesting short-lived camp sites and the processing of a range of foods.

All necessary permits were obtained for the excavations and sample analysis for this study, which complied with all relevant regulations as set out by the Commission for Tourism and National Heritage (SCTH).

### Radiocarbon dating

At each site, a 1 m or 2 m wide trench was excavated from the edge of the site to the centre at what was judged to be the deepest point, in order to expose a section across one half of the mound and through the full depth of the deposit to the underlying natural surface. The exposed sections were cleaned, photographed, and drawn, with particular attention to evidence of layering, ash lenses, and changes in shell composition and/or condition. Columns of bulk samples measuring 20 cm x 20 cm in area were extracted from the section in 5 cm spits constrained by layer boundaries for quantitative shell analysis. Individual samples of shell or charcoal for dating were removed directly from the section in relation to the observed stratigraphy. Also, paired samples of terrestrial (charcoal) and marine (shell) samples were collected from layers that showed no signs of intermixing or fragmentation to provide new information on the local marine reservoir effect.

Radiocarbon dates were measured at the Oxford Radiocarbon Accelerator Unit (University of Oxford), using routine pretreatment protocols (phosphoric acid hydrolysis). In total 55 samples were measured, of which 49 were shells and 6 were charcoal. For each site, we selected one dating sample from the uppermost shell layer, one from the lowermost layer, and at least one from an intermediate layer depending on the depth of the deposit. At some sites we found shells embedded in the underlying soil or beach ridge and we dated these too in order to give a *terminus post quem* for the start of human shellfish collection.

Previous analyses of radiocarbon dates from the Farasan shell middens have used varying ΔR values to correct for the local marine reservoir effect– ΔR = 100±50 [64,67] and ΔR = 123±28 [68]. Here we report the addition of four paired samples of terrestrial charcoal and marine shell from the sites of JE0087, JW1727 and JW1807, which produced values of 48±32, 73±47, 154±65 and 188±44. We tested the differences in modelled dates using the smallest and largest ΔR values and found that they do not significantly change our models. To best account for pronounced local variations, we selected the largest ΔR value (188±44) for all modelled results. Finally, we used Bayesian analysis in OxCal and its *Interval* query to refine estimates of duration for a group of deposition events and thus to further constrain the rates of shell accumulation at individual sites.

## Results

### Chronological framework for midden sites

**Fig 10:**
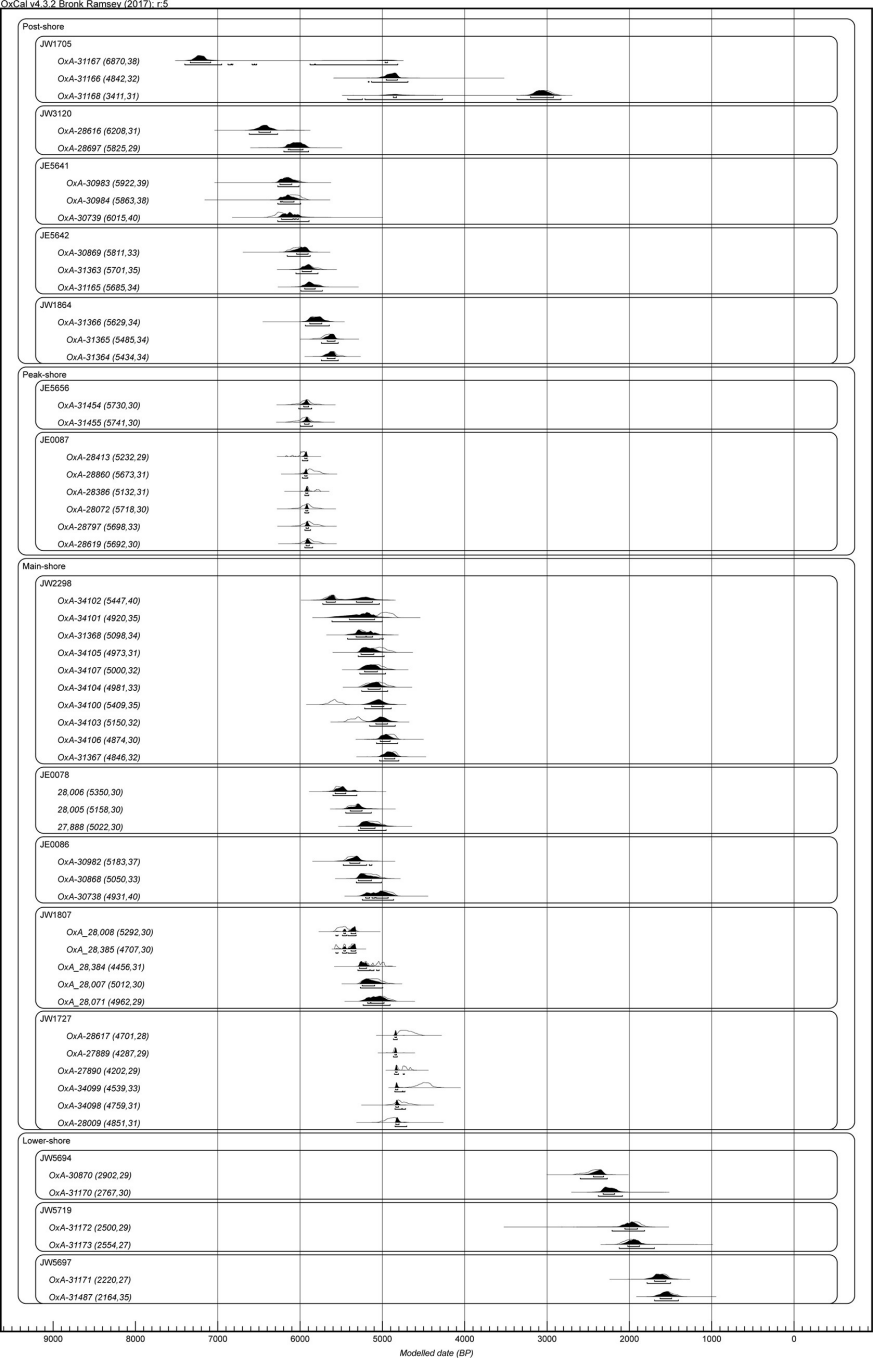
All radiocarbon dates grouped chronologically and by spatial group. Dating convention as in Table 1.

The full results are set out in Table 1 and Fig 10 Dates are modelled in OxCal (4.3.2) using marine or terrestrial calibration curves (Marine 13/Intcal13) according to type of dated material and are expressed as calibrated BP dates with 95.4% confidence intervals (see also S1 Table).

**Table 1:** Radiocarbon dates used for calculating the time depth and accumulation rates for all sites. Dates are shown as calibrated years BP (95.4% confidence interval) following Bronk Ramsey[69]. All dates are calibrated using Oxcal (version 4.3.2) and the corresponding curves for terrestrial (IntCal13) or marine (Marine13) samples as appropriate [70] with a local reservoir correction of 188±44 years. Sites are grouped by type of location, within each location type in order of earliest deposits, and within each site by stratigraphic order from lowest to highest deposits.

Site	Laboratory Code	^14^C-Age	±	MRE	Calibrated BP (95.4%)		Material	Species		Depth [m]
**Post-shore**										
JW1705	OxA-31167	6870	38	188±44	7360	7030	marine shell	*C*. *fasciatus*		1.00
	OxA-31166	4842	32	188±44	5050	4710	marine shell	*C*. *fasciatus*		0.55
	OxA-31168	3411	31	188±44	3370	2820	marine shell	*C*. *fasciatus*		0.10
JW3120	OxA-28616	6208	31	188±44	6590	6290	marine shell	*C*. *fasciatus*		0.65
	OxA-28697	5825	29	188±44	6200	5900	marine shell	*C*. *fasciatus*		0.10
JE5641	OxA-30983	5922	39	188±44	6270	6010	marine shell	*C*. *fasciatus*		0.20
	OxA-30984	5863	38	188±44	6270	5990	marine shell	*C*. *fasciatus*		0.20
	OxA-30739	6015	40	188±44	6270	5920	marine shell	*C*. *fasciatus*		0.05
JE5642	OxA-30869	5811	33	188±44	6160	5870	marine shell	*C*. *fasciatus*		0.60
	OxA-31363	5701	35	188±44	6050	5780	marine shell	*C*. *fasciatus*		0.30
	OxA-31165	5685	34	188±44	6000	5730	marine shell	*C*. *fasciatus*		0.05
JW1864	OxA-31366	5629	34	188±44	5940	5630	marine shell	*C*. *fasciatus*		1.15
	OxA-31365	5485	34	188±44	5830	5550	marine shell	*C*. *fasciatus*		0.05
	OxA-31364	5434	34	188±44	5750	5480	marine shell	*C*. *fasciatus*		0.05
**Peak-shore**										
JE5656	OxA-31454	5730	30	188±44	6010	5860	marine shell	*C*. *fasciatus*		0.40
	OxA-31455	5741	37	188±44	5990	5850	marine shell	*C*. *fasciatus*		0.10
JE0087	OxA-28413	5232	29		5970	5900	charcoal	n/a		1.35
	OxA-28860	5673	31	188±44	5970	5900	marine shell	*C*. *fasciatus*		1.35
	OxA-28386	5132	31		5950	5890	charcoal	n/a		1.00
	OxA-28072	5718	30	188±44	5950	5890	marine shell	*C*. *fasciatus*		1.00
	OxA-28797	5698	33	188±44	5950	5870	marine shell	*C*. *fasciatus*		0.00
	OxA-28619	5692	30	188±44	5950	5850	marine shell	*C*. *fasciatus*		0.05
**Main-shore**										
JW2298	OxA-34102	5447	40	188±44	5710	5040	marine shell	*C*. *fasciatus*		1.85
	OxA-34101	4920	35	188±44	5580	5040	marine shell	*C*. *fasciatus*		1.65
	OxA-31368	5098	34	188±44	5400	5030	marine shell	*C*. *fasciatus*		1.50
	OxA-34105	4973	31	188±44	5290	5010	marine shell	*C*. *fasciatus*		1.30
	OxA-34107	5000	32	188±44	5270	4990	marine shell	*C*. *fasciatus*		1.20
	OxA-34104	4981	33	188±44	5240	4960	marine shell	*C*. *fasciatus*		0.95
	OxA-34100	5409	35	188±44	5210	4910	marine shell	*C*. *fasciatus*		0.60
	OxA-34103	5150	32	188±44	5160	4870	marine shell	*C*. *fasciatus*		0.25
	OxA-34106	4874	30	188±44	5080	4830	marine shell	*C*. *fasciatus*		0.20
	OxA-31367	4846	32	188±44	5040	4810	marine shell	*C*. *fasciatus*		0.05
JE0078	OxA-28006	5350	30	188±44	5600	5300	marine shell	*C*. *fasciatus*		1.00
	OxA-28005	5158	30	188±44	5450	5130	marine shell	*C*. *fasciatus*		0.50
	OxA-27888	5022	30	188±44	5300	4950	marine shell	*C*. *fasciatus*		0.10
JE0086	OxA-30982	5183	37	188±44	5480	5190	marine shell	*C*. *fasciatus*		1.10
	OxA-30868	5050	33	188±44	5320	5010	marine shell	*C*. *fasciatus*		0.70
	OxA-30738	4931	40	188±44	5250	4860	marine shell	*C*. *fasciatus*		0.15
JW1807	OxA-28008	5292	29	188±44	5420	5310	marine shell	*C*. *fasciatus*		3.30
	OxA-28385	4707	31		5570	5310	charcoal	n/a		3.30
	OxA-28384	4456	30		5300	5040	charcoal	n/a		2.30
	OxA-28007	5012	30	188±44	5200	5000	marine shell	*Brachidontes*	sp.	0.70
	OxA-28071	4962	31	188±44	5240	4910	marine shell	*C*. *fasciatus*		0.10
JW1727	OxA-28617	4701	28	188±44	4870	4810	marine shell	*Brachidontes*	sp.	1.68
	OxA-27889	4287	29		4870	4810	charcoal	n/a		1.68
	OxA-27890	4202	29		4860	4730	charcoal	n/a		0.95
	OxA-34099	4539	33	188±44	4850	4720	marine shell	*C*. *fasciatus*		0.50
	OxA-34098	4759	31	188±44	4850	4710	marine shell	*C*. *fasciatus*		0.40
	OxA-28009	4851	31	188±44	4850	4700	marine shell	*Brachidontes*	sp.	0.15
**Low-shore**										
JW5694	OxA-30870	2902	29	188±44	2600	2260	marine shell	*C*. *fasciatus*		0.40
	OxA-31170	2767	30	188±44	2380	2070	marine shell	*C*. *fasciatus*		0.30
JW5719	OxA-31172	2500	29	188±44	2210	1810	marine shell	*C*. *fasciatus*		0.30
	OxA-31173	2554	27	188±44	2120	1750	marine shell	*C*. *fasciatus*		0.10
JW5697	OxA-31171	2220	27	188±44	1790	1500	marine shell	*Nerita* sp.		0.40
	OxA-31487	2164	35	188±44	1700	1400	marine shell	*C*. *fasciatus*		0.10

The earliest date for shellfish exploitation on Farasan is 7,360–7,030 cal BP (Table 1, Fig 10) from the post-shore site JW1705. In fact, all early dates come from the post-shore sites within our transects (JW1705, JW3120, JE5641, JE5642, JW1864).

The earliest dates for mounds sitting on the palaeoshorelines are the two sites on the peak shoreline in Transect 3 (JE5656 and JE0087), with dates respectively of 6,010–5,860 and 5,970–5,900 cal BP. This is consistent with our interpretation of this features as a shoreline associated with the Highstand maximum, which is independently dated at 6,000 cal BP. These sites are slightly earlier in date than those on the main shoreline in the Janaba East cluster. It should be noted that JE5656 is an extensive scatter about 20 m in diameter and not more than 50 cm thick with numerous unstratified potsherds and lithics. It may originally have been a more substantial mound but has numerous four-wheel-drive wheel tracks running over it, suggesting that it has been damaged by vehicle traffic. In any case, it appears that this is a habitation site rather than a specialised processing site, in contrast to the other shell mounds on the palaeoshorelines in this transect. It demonstrates that not all middens used as habitation sites are in post-shore locations and not all shoreline deposits are processing sites.

The dates for the high concentration of middens on the main shoreline show that they ended around the same time, i.e. 5,300–4,950 cal BP (JE0078); 5,250–4,860 cal BP (JE0086); 5,040–4,810 cal BP (JW2298); 5,240–4,910 cal BP (JW1807) and 4,850–4,700 cal BP (JW1727). We take the latest dates of those sites to represent the end of large-scale shellfish exploitation on Farasan.

A series of later dates come from the low-shore middens, i.e. 2,600–2,070 cal BP (JW5694); 2,210–1,750 cal BP (JW5719); and 1,790–1,400 cal BP (JW5697). The small number of these sites and their more mixed composition, with relatively small numbers of shells, and a relatively large proportion of mammal and fish bone suggests that they formed under different economic circumstances and are not related to the other sites in the cluster. They also indicate a marked reduction in the scale of shellfishing.

### Deposition intervals and deposition rates

Table 2 shows that there is a large variation in duration; some deposits accumulated over a period of as much as 3,820 years (JW1705) or as little as 88 years (JW1727) (both 95.4% confidence interval) (see also S1 File). These differences are not correlated with the size of the midden, as would be the case if the rate of shell accumulation was uniform between sites. The shallow post-shore middens range from 320 to 3,820 years in duration, suggesting relatively slow rates of shell deposition, while the deeper shoreline mounds range from 88 to 1,466 years and thus have slightly shorter durations on average than the shallow middens and faster rates of shell accumulation.

**Table 2:** Depth of deposit and accumulation rates in m per thousand years (ka) based on the difference between depths of lowest and highest radiocarbon samples within each site.

Group	Site	Interval (95.4%)	Depth[m]	Accumulation Rate [m/ka]
Post-shore	JW1705	3,820	0.9	0.24
	JE5641	320	0.15	0.47
	JW3120	1,055	0.55	0.52
	JE5642	717	0.55	0.77
	JW1864	850	1.1	1.29
Lower-shoreline	JW5694	904	0.1	0.11
	JW5719	744	0.2	0.27
	JW5697	738	0.3	0.41
Peak and main shoreline	JE5656	688	0.3	0.44
	JE0086	1,466	0.95	0.65
	JE0078	1,129	0.9	0.8
	JW1807	644	3.2	4.97
	JW2298	291	1.8	6.19
	JE0087	122	1.3	10.66
	JW1727	88	1.53	17.39

**Fig 11:**
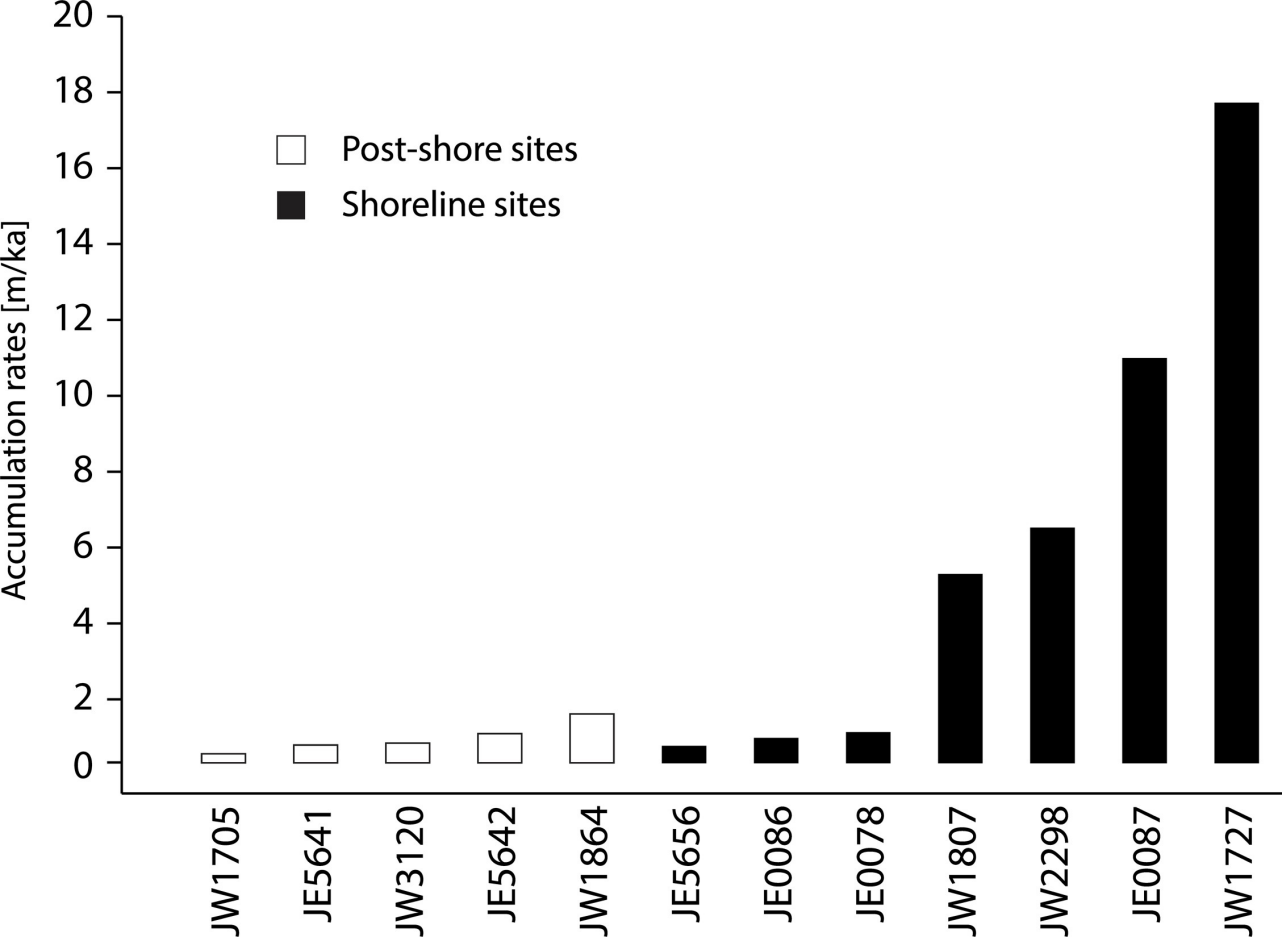
Accumulation rates ordered by site type. Exact rates are shown in Table 2. ‘Lower’ shoreline sites (JW5694, JW5719, JW5697) are excluded as they fall outside the main phase of shellfish gathering.

The rates of accumulation of shoreline and post-shore sites are distinctly different (Table 2; Fig 11). Shoreline sites show a range of accumulation rates of 0.24–1.29 m/ka (mean = 0.66 m/ka), while the range at processing sites is 0.11–17.39 m/ka (mean = 4.19 m/ka). The rate at processing sites is higher still– 5.87 m/ka if we exclude the youngest sites from the lower shorelines and include only those sites that fall within the main period of shellfish exploitation (i.e. c. 7,360 to 4,700 cal BP). In other words, the rate of accumulation of shells in the shoreline sites is roughly ten times the rate in post-shore sites.

This difference in rates of accumulation is consistent with our original expectations and suggests that the majority of shellfish processing and subsequent accumulation of shells was indeed carried out on the shoreline and that whole shellfish (meat and shell) were transported inland to post-shore sites in much smaller quantities. Furthermore, we might expect that the further inland the post-shore site, the lower the rate of shell accumulation. However, there does not appear to be any correlation within the post-shore group between distance from the shore and rate of accumulation. For instance, JW1705 and JW1864, the post-shore sites with the lowest and highest accumulation rates respectively, are at the same distance from their contemporaneous shoreline. The implication is that there is not a simple linear relationship between rates of accumulation and distance from the shoreline. One factor that may be relevant here is that JW1705 with occupation over a longer period and low rates of accumulation is dominated by shells of the large mollusc *Chicoreus ramosus*, whereas JW1864 with a shorter duration and a high rate of accumulation is dominated by the much smaller gastropod *C*. *fasciatus*. These shells may give rise to different rates of accumulation and may also impose different constraints on the distances over which they can conveniently be transported. However, we do not have enough sites to explore further the relationship between shell composition and transportation distance. The important general point is that the rate of accumulation at post-shore sites is generally lower than at shoreline sites.

Although the rate of accumulation in shoreline sites is generally higher than for post-shore sites, there is, nevertheless, a wide range of variation within the shoreline group. This suggests that they are not all formed under the same conditions, and there are likely other underlying factors influencing how often different shoreline sites were revisited. Also, seasonality data available for JW1727 suggest that individual layers are composites of several visits throughout the year with varying frequency [71]. Thus, it is unsurprising that we find some variability in the deposition rates of shell mounds in similar types of location and with seemingly similar morphologies. These differences might reflect such variables as the different quantity of molluscs available at different times within the overall duration of the midden or different numbers of people involved in shell food processing at different times or in different locations. It might also reflect differences in taxonomic composition as suggested above in relation to the post-shore sites. Many sites on shorelines and in post-shore positions have layers dominated by the large gastropod *C*. *ramosus* alternating with layers dominated by the small *C*. *fasciatus*, and these two species may generate different rates of shell consumption and discard. A more detailed programme of radiocarbon dating would be needed to unravel this pattern.

Lastly, the occurrence of high accumulation rates in shoreline mounds is consistent with the absence of habitational or hearth structures in most of these sites, in contrast to the post-shore sites. This strengthens our inference that the primary use of shoreline sites was for the processing of shellfish immediately after collection, although we have noted exceptions above, as in the case of JE5656 in the Janaba East cluster.

## Discussion

### Differential preservation and sea level change

Our results show that for the first ~1,360 years (~7,360–6,000 cal BP) relatively few shells were accumulated, as only post-shore sites date to this period. Only with the occurrence of shoreline sites at 5,970–5,900 cal BP (OxA-28,413, JE0087) do accumulation rates rise dramatically. This trend continues with the sites of JW1807, JW2298 and JW1727 until 4,850–4,700 cal BP (OxA-28,009, JW1727). If we were to take this evidence at face value, we would infer that there was a major intensification of shellfishing after 6,000 cal BP.

However, the alternative hypothesis is that the change is the result of a change in the preservation and visibility of the processing sites. The apparent economic shift from small-scale shell processing in post-shore sites at ~7,360 cal BP, to the coeval occurrence of small-scale processing at post-shore sites and large-scale processing on the shoreline at ~6,000 cal BP and later makes sense when compared with the evidence for a Highstand at ~6,000 cal BP and the subsequent drop in sea level. This confirms our prediction that the earliest dated sites are post-shore ones. We conclude that if processing sites existed between 7,360 and 6,000 cal BP, they have been lost because of inundation by sea-level rise. It is, of course, possible that processing sites did not exist in this earlier period because molluscs were not available in sufficient quantity to generate high rates of shell accumulation and large shell mounds at shoreline sites, or because people chose not to exploit the molluscs in this period despite their availability and only collected small quantities that were easily carried back in their entirety to post-shore sites before being processed. We cannot decisively rule out those possibilities. However, we consider them unlikely. The geomorphology of shallow bays with abundant molluscs would have existed in the 1,300 years prior to the Highstand as it did for a period of at least 2,500 after the marine regression following the Highstand, and people were clearly interested in exploiting molluscs before the Highstand as is demonstrated by their processing of small numbers of molluscs at post-shore sites which include *C*. *fasciatus*, the mollusc that dominates the large shoreline mounds of the later period. We also emphasise that post-shore sites continued in use after the Highstand, demonstrating a spatial separation between habitation sites and processing sites in the later period. We see no reason to suppose that a similar spatial separation of activities did not exist before the Highstand.

We also draw attention to the existence of a shallow deposit of *C*. *fasciatus* shells associated with a hearth dated to 6,500 cal BP [68] (S1 File). This deposit was found beneath the thick beach ridge on which the shell mound of JE0087 in Transect 3 is located. It was too shallow for the use of radiocarbon dates to measure the rate of accumulation. Nevertheless, it is clear evidence for the collection of *C*. *fasciatus* before the Highstand at a time when the palaeoshoreline was presumably very close. Evidently it has survived the impact of wave action during the Highstand because it was quickly buried beneath marine sand and was further sealed in stratigraphic position by the subsequent accumulation of a shell mound on the same spot. It provides a hint of what may be missing from the period before the Highstand, and also an indication of the conditions in which deposits accumulated during periods of lower sea level may have been preserved in the face of the potentially destructive impact associated with inundation by sea-level rise.

These results present an interesting paradox. Post-shore sites, for the most part, because of their relatively low rates of shell accumulation are more vulnerable to in situ damage, degradation and deflation by post-depositional effects such as trampling and physical and chemical degradation by weathering and exposure, and this is readily apparent from our field observations. However, because of their inland position, they are better protected from destruction by sea-level rise or erosion at the shore edge. Conversely, processing sites, because of the more rapid accumulation of shells, are less vulnerable to the processes of in situ damage, but because of their location on the shore edge they are more exposed to loss by sea level rise.

With a lateral movement of the shoreline between 7,360 and 6,000 cal BP of 100–200 m inland in the shallowest areas, the taphonomic impact on shoreline sites would have been considerable. The scope of destruction can be illustrated by considering the large number of sites (~1,700) that are now visible on post-Highstand shorelines. If the bays in front of these sites were as productive of shellfish before the Highstand as during and after the period of the Highstand, then a very substantial volume of midden deposits has been lost. Had we taken the surviving evidence of post-shore shell-midden deposits in the period before the Highstand at face value as an accurate representation of shellgathering in this early period, we would have seriously understated the role of shell food and produced estimates of shell quantities an order of magnitude lower than we believe to have been the case.

### Wider implications

Our results have more general implications for the investigation of groups of shell mounds in other parts of the world, and especially for sites that are associated with periods of lower sea level. Clusters of Holocene shell middens that include post-shore/habitational sites have been reported in other parts of the world [19,72–74], in some cases in clear association with changes of sea level or shoreline development, and we suggest that these would repay further investigation in the light of the results from the Farasan.

There is also the more general problem of larger-scale changes in shorelines and site visibility associated with the glacial-interglacial cycle of sea-level change. That shell middens could have existed during periods in the early Holocene and late Pleistocene when sea-level was substantially lower than the present but have been inundated by sea level rise and washed away or buried under marine sediment is well recognised [22,41,75,76]. The additional insight that we bring to this discussion is that it is not only shell middens in general that are likely to be lost by sea-level rise, but a specific class of shell middens, i.e. shell processing sites, which are likely to be under-represented in the record because of their proximity to the shoreline. Previous studies have provided plausible grounds for raising that possibility in the interpretation of early Holocene and late Pleistocene coastal sites that contain varying quantities of shells in deeply stratified deposits that span periods of late Pleistocene sea-level change. In these cases, the relative quantities of shells vary with the varying distance between the cave and the shoreline, increasing in quantity as the shoreline moved closer to the cave during periods of rising sea level and decreasing as sea level dropped and the shoreline moved away [41]. The implication is that during periods of lower sea level shells were deposited at sites closer to the shoreline that are now missing from the archaeological record. What we provide in the Farasan example is a quantitative demonstration of the difference in shell quantities and rates of accumulation between post-shore and shoreline sites and evidence for the presence of the missing sites that likely existed when sea level was lower than the present.

Many coastal caves and rockshelters around the coastlines of southern Europe, the Mediterranean and Africa have been reported with mollusc shells as food remains in varying quantities in early Holocene and late Pleistocene deposits extending as far back as the Last Interglacial (MIS 5) and the preceding glacial period of low sea level (MIS 6) [77–85]. Increasingly, interest has focused on these shell deposits as proxy indicators of developing human cognitive capacities, dispersal patterns, economic intensification or population growth [86–89] and often these studies employ quantitative measurements of some kind (e.g. shell density, shell accumulation rates, or shell weight) [89–91]. In the light of the Farasan case study reported here, we contend that the shell deposits in these sites, and especially those associated with lower sea levels, are very likely to be the equivalent of our post-shore sites, which, because of their distance from the contemporaneous shoreline or their elevation above it, contain only a fraction of the total shell food collected during any interval of time. The large remainder is likely to have been processed or consumed at locations closer to the mollusc supply and therefore more vulnerable to removal from the archaeological record by erosion or submergence. In this taphonomic context the application of quantitative shell measures will thus fail to provide accurate proxy information.

We think this is likely to apply even to those cave sites where the shells were deposited during periods of high sea level such as the Last Interglacial (Marine Isotope stage 5e at 125,000 years ago) or in those rare cases of subducting coastlines, as in Timor and the Bismarck archipelago [92,93] where coastal caves have always remained close to the present shoreline because of a steep offshore profile and a rate of tectonic uplift that has kept pace with the Late Glacial rise in sea level [94]. Our reason for thinking this is that, even in these examples, the caves would have remained at some distance inland from the shore and elevated to some degree above it. The distances and elevations might have been quite small, but the labour costs of transporting molluscs in the shell are likely to have been sensitive even to small extra increments of effort involved in their collection and transportation and reinforced the preference for processing as many shells as possible as close as possible to the shoreline.

### Conclusions

By combining statistical analysis of multiple radiocarbon dates with shell quantities across a range of shell-midden deposits, we have been able to demonstrate considerable inter-site variation in rates of shell accumulation. The highest rates of shell accumulation occur in the largest mounds and show that in some cases substantial deposits accumulated within a matter of decades. There is also a significant difference between rates of accumulation in deposits situated on shorelines immediately adjacent to the source of the live molluscs and deposits situated further inland, which we describe as post-shore sites. These post-shore sites are located at a short distance inland from the contemporaneous shoreline and we interpret them as habitation sites where location was determined primarily by access to resources other than shellfish, such as shelter, terrestrial plant and animal foods, and water supplies. The highest rates of accumulation and the largest deposits occur on the shoreline, while the volume of shell material in post-shore deposits is generally much smaller and the rate of shell accumulation lower by an order of magnitude than in the shoreline sites. We attribute this difference to the high labour costs of transporting marine molluscs in the shell and the preference for processing molluscs to remove the shell as close as possible to the source of supply. We interpret the shoreline deposits as specialized sites used primarily for processing molluscs and perhaps also for their consumption, in contrast to the post-shore sites, and support this difference in function with reference to the limited presence of artefacts or other food remains in the shoreline sites, in contrast to the post-shore sites. The latter sites also have evidence for the presence of stone-lined hearths and remains of habitation structures built of coral or beachrock, suggesting longer-term occupation consistent with interpretation as habitation sites. A systematic comparison of artefacts and non-shell faunal remains from different sites to further explore differences in site function, as has been carried out elsewhere (e.g. [95] has not proved possible in the Farasan case because of the high rates of shell accumulation, the low density of non-shell remains and the relatively small volumes of deposit excavated. Nevertheless, we have been able to use such remains as are present in excavation together with surface indications to corroborate some of our inferences about differences in site function as well as to identify sites that do not fit the expected pattern, for example the case of JE5656, which is a shoreline site that appears to have the characteristics of a habitation site rather than a processing site. Seasonality analyses of shells offers another potential avenue of investigation for identifying differences in site function [96]. An important conclusion that emerges from our analysis is the importance of inter-site comparisons and the need to factor into the interpretation of individual shell-midden deposits the likelihood that they represent only one part of a wider pattern of site use and cannot be assumed to fully represent the overall pattern. Moreover, the function of individual sites is likely to remain difficult to identify without such inter-site comparisons.

The shoreline and post-shore sites overlap in time from about 6,000 years ago, indicating that they represent different facets of the same settlement and subsistence system. In the period from c. 7,300 to 6,000 years ago, only post-shore sites are present, and we attribute the absence of processing sites in this period to the fact that sea level was lower at this time and that any processing sites situated on the shorelines of this period have been removed by inundation and erosion as sea level rose by c. 2 m to reach a mid-Holocene Highstand at c. 6,000 years ago, with the possible exception of a shallow shell scatter and hearth sealed beneath the sand ridge of the Highstand shoreline on which one of the larger shell mounds subsequently accumulated.

We further contend that the many coastal caves and rockshelters with deposits of food shells dating to periods of lower sea-level during the late Pleistocene and early Holocene on many of the world’s coastlines are post-shore sites, and that interpretations of the role of shell food at these sites and their significance as proxies for other developments in human behaviour are likely to be quite misleading, without taking into account the inter-site variations and the differences of site visibility and preservation that we have identified in the Farasan case study, especially the likely impact of sea-level change. The widespread impact of sea-level change in creating large gaps in the prehistoric coastal record is inescapable. Whether processing sites on or close to the shore, or indeed post-shore sites that have been inundated by a more substantial rise in sea level, could have survived the effects of marine erosion and submergence remains uncertain. Preliminary underwater searches for shell midden deposits on submerged palaeoshorelines in the Farasan Islands have so far proved unsuccessful [61,97]. On the other hand, recent research in Denmark and the Gulf of Florida has demonstrated that substantial shell-midden deposits can survive submergence in favourable circumstances and be investigated using underwater techniques [98,99] and new investigations in various parts of the world are beginning to open up the exploration of submerged landscapes. The search for the missing palaeoshorelines of the Last Glacial period and their associated coastal sites remains one of the great challenges for the future. High-resolution studies of mid-to-late Holocene shell mounds, where it is possible to exercise some control on variables of differential preservation, transportation distance and changes of sea-level and coastal geomorphology, have an important role to play in refining models for the interpretation of Pleistocene evidence for shell food exploitation.

## Supporting information

S1 Table Radiocarbon dates for all sites and underlying layers. (XLSX)

S1 File Radiocarbon models for OxCal (4.3.2). (DOCX)
