# Correction: Quantifying spatial variability in shell midden formation in the Farasan Islands, Saudi Arabia

**DOI:** 10.1371/journal.pone.0303717

**Published:** 2024-05-09

**Authors:** Niklas Hausmann, Matthew Meredith-Williams, Katerina Douka, Robyn H. Inglis, Geoff Bailey

There is an error in Tables 1 and 2. Specifically, the radiocarbon date OxA-31167 is incorrectly included in some of the models and the results are affected. The depth value of 1.0 m in Table 1 should be written as “other column.” The resulting occupation interval for the site JW1705 in Table 2 should be changed from 3,820 to 1,980 (77.9%) and from 0.24 to 0.23. Please see the correct the tables and captions here.

**Table 1:** Radiocarbon dates used for calculating the time depth and accumulation rates for all sites. Dates are shown as calibrated years BP (95.4% confidence interval) following Bronk Ramsey[69]. All dates are calibrated using Oxcal (version 4.3.2) and the corresponding curves for terrestrial (IntCal13) or marine (Marine13) samples as appropriate [70] with a local reservoir correction of 188±44 years. Sites are grouped by type of location, within each location type in order of earliest deposits, and within each site by stratigraphic order from lowest to highest deposits.

Site	Laboratory Code	^14^C-Age	±	MRE	Calibrated BP (95.4%)		Material	Species	Depth [m]
**Post-shore**									
JW1705	OxA-31167	6870	38	188±44	7360	7030	marine shell	*C*. *fasciatus*	other column
	OxA-31166	4842	32	188±44	5050	4710	marine shell	*C*. *fasciatus*	0.55
	OxA-31168	3411	31	188±44	3370	2820	marine shell	*C*. *fasciatus*	0.10
JW3120	OxA-28616	6208	31	188±44	6590	6290	marine shell	*C*. *fasciatus*	0.65
	OxA-28697	5825	29	188±44	6200	5900	marine shell	*C*. *fasciatus*	0.10
JE5641	OxA-30983	5922	39	188±44	6270	6010	marine shell	*C*. *fasciatus*	0.20
	OxA-30984	5863	38	188±44	6270	5990	marine shell	*C*. *fasciatus*	0.20
	OxA-30739	6015	40	188±44	6270	5920	marine shell	*C*. *fasciatus*	0.05
JE5642	OxA-30869	5811	33	188±44	6160	5870	marine shell	*C*. *fasciatus*	0.60
	OxA-31363	5701	35	188±44	6050	5780	marine shell	*C*. *fasciatus*	0.30
	OxA-31165	5685	34	188±44	6000	5730	marine shell	*C*. *fasciatus*	0.05
JW1864	OxA-31366	5629	34	188±44	5940	5630	marine shell	*C*. *fasciatus*	1.15
	OxA-31365	5485	34	188±44	5830	5550	marine shell	*C*. *fasciatus*	0.05
	OxA-31364	5434	34	188±44	5750	5480	marine shell	*C*. *fasciatus*	0.05
**Peak-shore**									
JE5656	OxA-31454	5730	30	188±44	6010	5860	marine shell	*C*. *fasciatus*	0.40
	OxA-31455	5741	37	188±44	5990	5850	marine shell	*C*. *fasciatus*	0.10
JE0087	OxA-28413	5232	29		5970	5900	charcoal	n/a	1.35
	OxA-28860	5673	31	188±44	5970	5900	marine shell	*C*. *fasciatus*	1.35
	OxA-28386	5132	31		5950	5890	charcoal	n/a	1.00
	OxA-28072	5718	30	188±44	5950	5890	marine shell	*C*. *fasciatus*	1.00
	OxA-28797	5698	33	188±44	5950	5870	marine shell	*C*. *fasciatus*	0.00
	OxA-28619	5692	30	188±44	5950	5850	marine shell	*C*. *fasciatus*	0.05
**Main-shore**									
JW2298	OxA-34102	5447	40	188±44	5710	5040	marine shell	*C*. *fasciatus*	1.85
	OxA-34101	4920	35	188±44	5580	5040	marine shell	*C*. *fasciatus*	1.65
	OxA-31368	5098	34	188±44	5400	5030	marine shell	*C*. *fasciatus*	1.50
	OxA-34105	4973	31	188±44	5290	5010	marine shell	*C*. *fasciatus*	1.30
	OxA-34107	5000	32	188±44	5270	4990	marine shell	*C*. *fasciatus*	1.20
	OxA-34104	4981	33	188±44	5240	4960	marine shell	*C*. *fasciatus*	0.95
	OxA-34100	5409	35	188±44	5210	4910	marine shell	*C*. *fasciatus*	0.60
	OxA-34103	5150	32	188±44	5160	4870	marine shell	*C*. *fasciatus*	0.25
	OxA-34106	4874	30	188±44	5080	4830	marine shell	*C*. *fasciatus*	0.20
	OxA-31367	4846	32	188±44	5040	4810	marine shell	*C*. *fasciatus*	0.05
JE0078	OxA-28006	5350	30	188±44	5600	5300	marine shell	*C*. *fasciatus*	1.00
	OxA-28005	5158	30	188±44	5450	5130	marine shell	*C*. *fasciatus*	0.50
	OxA-27888	5022	30	188±44	5300	4950	marine shell	*C*. *fasciatus*	0.10
JE0086	OxA-30982	5183	37	188±44	5480	5190	marine shell	*C*. *fasciatus*	1.10
	OxA-30868	5050	33	188±44	5320	5010	marine shell	*C*. *fasciatus*	0.70
	OxA-30738	4931	40	188±44	5250	4860	marine shell	*C*. *fasciatus*	0.15
JW1807	OxA-28008	5292	29	188±44	5420	5310	marine shell	*C*. *fasciatus*	3.30
	OxA-28385	4707	31		5570	5310	charcoal	n/a	3.30
	OxA-28384	4456	30		5300	5040	charcoal	n/a	2.30
	OxA-28007	5012	30	188±44	5200	5000	marine shell	*Brachidontes sp*.	0.70
	OxA-28071	4962	31	188±44	5240	4910	marine shell	*C*. *fasciatus*	0.10
JW1727	OxA-28617	4701	28	188±44	4870	4810	marine shell	*Brachidontes sp*.	1.68
	OxA-27889	4287	29		4870	4810	charcoal	n/a	1.68
	OxA-27890	4202	29		4860	4730	charcoal	n/a	0.95
	OxA-34099	4539	33	188±44	4850	4720	marine shell	*C*. *fasciatus*	0.50
	OxA-34098	4759	31	188±44	4850	4710	marine shell	*C*. *fasciatus*	0.40
	OxA-28009	4851	31	188±44	4850	4700	marine shell	*Brachidontes sp*.	0.15
**Low-shore**									
JW5694	OxA-30870	2902	29	188±44	2600	2260	marine shell	*C*. *fasciatus*	0.40
	OxA-31170	2767	30	188±44	2380	2070	marine shell	*C*. *fasciatus*	0.30
JW5719	OxA-31172	2500	29	188±44	2210	1810	marine shell	*C*. *fasciatus*	0.30
	OxA-31173	2554	27	188±44	2120	1750	marine shell	*C*. *fasciatus*	0.10
JW5697	OxA-31171	2220	27	188±44	1790	1500	marine shell	*Nerita* sp.	0.40
	OxA-31487	2164	35	188±44	1700	1400	marine shell	*C*. *fasciatus*	0.10

**Table 2:** Depth of deposit and accumulation rates in m per thousand years (ka) based on the difference between depths of lowest and highest radiocarbon samples within each site. Intervals have 95.4% confidence unless indicated otherwise.

Group	Site	Interval	Depth [m]	Accumulation Rate [m/ka]
Post-shore	JW1705	1,980 (77.9%)	0.45	0.23
	JE5641	320	0.15	0.47
	JW3120	1,055	0.55	0.52
	JE5642	717	0.55	0.77
	JW1864	850	1.1	1.29
Lower-shoreline	JW5694	904	0.1	0.11
	JW5719	744	0.2	0.27
	JW5697	738	0.3	0.41
Peak and main shoreline	JE5656	688	0.3	0.44
	JE0086	1,466	0.95	0.65
	JE0078	1,129	0.9	0.8
	JW1807	644	3.2	4.97
	JW2298	291	1.8	6.19
	JE0087	122	1.3	10.66
	JW1727	88	1.53	17.39

In the Deposition intervals and deposition rates subsection of Results, there is an error in the first and second paragraph.

The correct paragraphs should have read: Table 2 shows that there is a large variation in duration; some deposits accumulated over a period of as much as 1,980 years (JW1705; 77.9% confidence interval), with other parts of the midden suggesting even longer occupation, or as little as 88 years (JW1727; 95.4% confidence interval) (see also S1 File). These differences are not correlated with the size of the midden, as would be the case if the rate of shell accumulation was uniform between sites. The shallow post-shore middens range from 320 to 3,820 years in duration, suggesting relatively slow rates of shell deposition, while the deeper shoreline mounds range from 88 to 1,466 years and thus have slightly shorter durations on average than the shallow middens and faster rates of shell accumulation.

The rates of accumulation of shoreline and post-shore sites are distinctly different (Table 2; Fig 11). Post-shore sites show a range of accumulation rates of 0.23–1.29 m/ka (mean = 0.66 m/ ka), while the range at shoreline sites is 0.11–17.39 m/ka (mean = 4.19 m/ka). The rate at shoreline sites is higher still– 5.87 m/ka if we exclude the youngest sites from the lower shorelines and include only those sites that fall within the main period of shellfish exploitation (i.e. c. 7,360 to 4,700 cal BP). In other words, the rate of accumulation of shells in the shoreline sites is roughly ten times the rate in post-shore sites.
