# Supplementary material for: Quantifying spatial variability in shell midden formation in the Farasan Islands, Saudi Arabia
Source: PLoS One. 2019 Jun 12;14(6):e0217596. doi: 10.1371/journal.pone.0217596 (PMC6561681; doi:10.1371/journal.pone.0217596)
Supplement: S1 File — (DOCX) [file pone.0217596.s002.docx]

## Supplementary Material 2 – Radiocarbon Models for OxCal (4.3.2)

Quantifying Spatial Variability in Shell Midden Formation in the Farasan Islands, Saudi Arabia

Niklas Hausmann^1,2*^, Matthew Meredith-Williams^2,3^, Katerina Douka^1^, Robyn Inglis^2^, Geoff Bailey^2^,^4^

1 Max-Planck-Institute for the Science of Human History, Jena, Germany
2 University of York, Heslington, United Kingdom
3 La Trobe University, Bundoora-Melbourne, Australia
4 Flinders University, Adelaide, Australia

*corresponding author: hausmann@shh.mpg.de

## Model for all sites ordered by group

Plot()

{

Outlier_Model("General",T(5),U(0,4),"t");

Phase("Farasan")

{

Sequence("Post-shore")

{

Boundary("Start Post-shore");

Phase()

{

Sequence("JW1705")

{

Boundary("Start JW1705");

Sequence()

{

Curve("Marine13","Marine13.14c");

Delta_R("LocalMarine",188,44);

R_Date("OxA-31167",6870,38)

{

z=1.00;

Outlier(0.05);

};

Boundary();

R_Date("OxA-31166",4842,32)

{

z=0.55;

Outlier(0.05);

};

Boundary();

R_Date("OxA-31168",3411,31)

{

z=0.1;

Outlier(0.05);

};

Interval("Interval");

};

Boundary("End JW1705");

};

Sequence("JW3120")

{

Boundary("Start JW3120");

Sequence()

{

Curve("Marine13","Marine13.14c");

Delta_R("LocalMarine",188,44);

R_Date("OxA-28616",6208,31)

{

z=0.65;

Outlier(0.05);

};

R_Date("OxA-28697",5825,29)

{

z=0.1;

Outlier(0.05);

};

Interval("Interval");

};

Boundary("End JW3120");

};

Sequence("JE5641")

{

Boundary("Start JE5641");

Sequence()

{

Curve("Marine13","Marine13.14c");

Delta_R("LocalMarine",188,44);

Phase("Layer 3")

{

R_Date("OxA-30983",5922,39)

{

z=0.2;

Outlier(0.05);

};

R_Date("OxA-30984",5863,38)

{

z=0.2;

Outlier(0.05);

};

};

R_Date("OxA-30739",6015,40)

{

z=0.05;

Outlier(0.05);

};

Interval("Interval");

};

Boundary("End JE5641");

};

Sequence("JE5642")

{

Boundary("Start JE5642");

Sequence()

{

Curve("Marine13","Marine13.14c");

Delta_R("LocalMarine",188,44);

R_Date("OxA-30869",5811,33)

{

z=0.6;

Outlier(0.05);

};

R_Date("OxA-31363",5701,35)

{

z=0.3;

Outlier(0.05);

};

R_Date("OxA-31165",5685,34)

{

z=0.05;

Outlier(0.05);

};

Interval("Interval");

};

Boundary("End JE5642");

};

Sequence("JW1864")

{

Boundary("Start JW1864");

Sequence()

{

Curve("Marine13","Marine13.14c");

Delta_R("LocalMarine",188,44);

R_Date("OxA-31366",5629,34)

{

z=1.15;

Outlier(0.05);

};

Phase("Layer 1")

{

R_Date("OxA-31365",5485,34)

{

z=0.05;

Outlier(0.05);

};

R_Date("OxA-31364",5434,34)

{

z=0.05;

Outlier(0.05);

};

};

Interval("Interval");

};

Boundary("End JW1864");

};

};

Boundary("End Post-shore");

};

Sequence("Peak Shoreline")

{

Boundary("Start Peak Shoreline");

Phase()

{

Sequence("JE0087")

{

Boundary("Start JE0087");

Sequence()

{

Combine("Layer 27")

{

Curve("IntCal13","IntCal13.14c");

R_Date("OxA-28413", 5232, 29)

{

Outlier(0.05);

z=1.35;

};

Curve("Marine13","Marine13.14c");

Delta_R("LocalMarine",188,44);

R_Date("OxA-28860",5673,31)

{

Outlier(0.05);

z=1.35;

};

};

Combine("Layer 14")

{

Curve("IntCal13","IntCal13.14c");

R_Date("OxA-28386", 5132, 31)

{

Outlier(0.05);

z=1;

};

Curve("Marine13","Marine13.14c");

Delta_R("LocalMarine",188,44);

R_Date("OxA-28072",5718,30)

{

Outlier(0.05);

z=1;

};

};

Curve("Marine13","Marine13.14c");

Delta_R("LocalMarine",188,44);

R_Date("OxA-28797",5698,33)

{

Outlier(0.05);

z=0.7;

};

Curve("Marine13","Marine13.14c");

Delta_R("LocalMarine",188,44);

R_Date("OxA-28619",5692,30)

{

Outlier(0.05);

z=0.25;

};

Interval("Interval");

};

Boundary("End JE0087");

};

Sequence("JE5656")

{

Boundary("Start JE5656");

Sequence()

{

Curve("Marine13","Marine13.14c");

Delta_R("LocalMarine",188,44);

R_Date("OxA-31454", 5730, 30);

R_Date("OxA-31455", 5741, 30);

Interval("Interval");

};

Boundary("End JE5656");

};

};

Boundary("End Peak Shoreline");

};

Sequence("Main Shoreline")

{

Boundary("Start Main Shoreline");

Phase()

{

Sequence("JE0078")

{

Boundary("Start JE0078");

Sequence()

{

Curve("Marine13","Marine13.14c");

Delta_R("LocalMarine",188,44);

R_Date("28,006", 5350, 30)

{

Outlier(0.05);

z=1.0;

};

R_Date("28,005", 5158, 30)

{

Outlier(0.05);

z=0.5;

};

R_Date("27,888", 5022, 30)

{

Outlier(0.05);

z=0.1;

};

Interval("Interval");

};

Boundary("End JE0078");

};

Sequence("JE0086")

{

Boundary("Start JE0086");

Sequence()

{

Curve("Marine13","Marine13.14c");

Delta_R("LocalMarine",188,44);

R_Date("OxA-30982",5183,37)

{

z=1.1;

Outlier(0.05);

};

R_Date("OxA-30868",5050,33)

{

z=0.7;

Outlier(0.05);

};

R_Date("OxA-30738",4931,40)

{

z=0.15;

Outlier(0.05);

};

Interval("Interval");

};

Boundary("End JE0086");

};

Sequence("JW1807")

{

Boundary("Start JW1807");

Sequence()

{

Combine("Layer 25/56")

{

Curve("Marine13","Marine13.14c");

Delta_R("LocalMarine",188,44);

R_Date("OxA_28,008", 5292, 30)

{

Outlier("General", 0.05);

z=3.3;

};

Curve("IntCal13","IntCal13.14c");

R_Date("OxA_28,385", 4707, 30)

{

Outlier("General", 0.05);

z=3.3;

};

};

Curve("IntCal13","IntCal13.14c");

R_Date("OxA_28,384", 4456, 31)

{

Outlier("General", 0.05);

z=2.3;

};

Curve("Marine13","Marine13.14c");

Delta_R("LocalMarine",188,44);

R_Date("OxA_28,007", 5012, 30)

{

Outlier("General", 0.05);

z=0.7;

};

R_Date("OxA_28,071", 4962, 29)

{

Outlier("General", 0.05);

z=0.1;

};

Interval("Interval");

};

Boundary("End JW1807");

};

Sequence("JW2298")

{

Boundary("Start JW2298");

Sequence()

{

Curve("Marine13","Marine13.14c");

Delta_R("LocalMarine",188,44);

R_Date("OxA-34102",5447,40)

{

z=1.85;

Outlier(0.05);

};

R_Date("OxA-34101",4920,35)

{

z=1.65;

Outlier(0.05);

};

R_Date("OxA-31368",5098,34)

{

z=1.5;

Outlier(0.05);

};

R_Date("OxA-34105",4973,31)

{

z=1.3;

Outlier(0.05);

};

R_Date("OxA-34107",5000,32)

{

z=1.2;

Outlier(0.05);

};

R_Date("OxA-34104",4981,33)

{

z=0.95;

Outlier(0.05);

};

R_Date("OxA-34100",5409,35)

{

z=0.6;

Outlier(0.05);

};

R_Date("OxA-34103",5150,32)

{

z=0.25;

Outlier(0.05);

};

R_Date("OxA-34106",4874,30)

{

z=0.2;

Outlier(0.05);

};

R_Date("OxA-31367",4846,32)

{

z=0.05;

Outlier(0.05);

};

Interval("Interval");

};

Boundary("End JW2298");

};

Sequence("JW1727")

{

Boundary("Start JW1727");

Sequence()

{

Combine()

{

Curve("Marine13","Marine13.14c");

Delta_R("LocalMarine",188,44);

R_Date("OxA-28617",4701,28)

{

z=1.68;

Outlier("General", 0.05);

};

Curve("IntCal13","IntCal13.14c");

R_Date("OxA-27889",4287,29)

{

Outlier("General", 0.05);

z=1.68;

};

};

Curve("IntCal13","IntCal13.14c");

R_Date("OxA-27890",4202,29)

{

Outlier("General", 0.05);

z=0.95;

};

Curve("Marine13","Marine13.14c");

Delta_R("LocalMarine",188,44);

R_Date("OxA-34099", 4539, 33)

{

Outlier("General", 0.05);

z=0.50;

};

Curve("Marine13","Marine13.14c");

Delta_R("LocalMarine",188,44);

R_Date("OxA-34098", 4759, 31)

{

Outlier("General", 0.05);

z=0.40;

};

Curve("Marine13","Marine13.14c");

Delta_R("LocalMarine",188,44);

R_Date("OxA-28009",4851,31)

{

Outlier("General", 0.05);

z=0.15;

};

Interval("Interval");

};

Boundary("End JW1727");

};

};

Boundary("End Main Shoreline");

};

Sequence("Lower Shorelines")

{

Boundary("Start Lower Shorelines");

Phase()

{

Sequence("JW5694")

{

Curve("Marine13","Marine13.14c");

Delta_R("LocalMarine",188,44);

R_Date("OxA-30870",2902,29)

{

z=0.4;

Outlier(0.05);

};

R_Date("OxA-31170",2767,30)

{

z=0.3;

Outlier(0.05);

};

Interval("Interval");

};

Sequence("JW5719")

{

Curve("Marine13","Marine13.14c");

Delta_R("LocalMarine",188,44);

R_Date("OxA-31172",2500,29)

{

z=0.3;

Outlier(0.05);

};

R_Date("OxA-31173",2554,27)

{

z=0.1;

Outlier(0.05);

};

Interval("Interval");

};

Sequence("JW5697")

{

Curve("Marine13","Marine13.14c");

Delta_R("LocalMarine",188,44);

R_Date("OxA-31171", 2220, 27)

{

Outlier(0.05);

};

R_Date("OxA-31487", 2164, 35)

{

z=0.1;

Outlier(0.05);

};

Interval("Interval");

};

};

Boundary("End Lower Shorelines");

};

};

};

## Depositional models by site

### JE0078

Plot()

{

Outlier_Model("General",T(5),U(0,4),"t");

Curve("Marine13","Marine13.14c");

Delta_R("LocalMarine",188,44);

Sequence("JE0078")

{

Boundary("Start 1");

Sequence("JE0078")

{

R_Date("28,006", 5350, 30)

{

Outlier(0.05);

z=1.0;

};

R_Date("28,005", 5158, 30)

{

Outlier(0.05);

z=0.5;

};

R_Date("27,888", 5022, 30)

{

Outlier(0.05);

z=0.1;

};

Interval("Interval of JE0078");

Span("Span of JE0078");

};

Boundary("End 1");

};

};

### JE0086

Plot()

{

Outlier_Model("G",T(5),U(0,4),"t");

Curve("Marine13","Marine13.14c");

Delta_R("LocalMarine",188,44);

Sequence("Farasan")

{

Boundary();

Sequence("Sequence of JE0086")

{

R_Date("OxA-30982",5183,37)

{

z=1.1;

Outlier(0.05);

};

R_Date("OxA-30868",5050,33)

{

z=0.7;

Outlier(0.05);

};

R_Date("OxA-30738",4931,40)

{

z=0.15;

Outlier(0.05);

};

Interval("Interval 0086");

Span("Span 0086");

};

Boundary();

};

};

### JE0087

Plot()

{

Outlier_Model("General",T(5),U(0,4),"t");

Sequence("JE0087")

{

Boundary("Start 1");

Sequence("JE0087")

{

Combine("Layer 27")

{

Curve("IntCal13","IntCal13.14c");

R_Date("OxA-28413", 5232, 29)

{

Outlier(0.05);

z=1.35;

};

Curve("Marine13","Marine13.14c");

Delta_R("LocalMarine",123,28);

R_Date("OxA-28860",5673,31)

{

Outlier(0.05);

z=1.35;

};

};

Combine("Layer 14")

{

Curve("IntCal13","IntCal13.14c");

R_Date("OxA-28386", 5132, 31)

{

Outlier(0.05);

z=1;

};

Curve("Marine13","Marine13.14c");

Delta_R("LocalMarine",123,28);

R_Date("OxA-28072",5718,30)

{

Outlier(0.05);

z=1;

};

};

Curve("Marine13","Marine13.14c");

Delta_R("LocalMarine",123,28);

R_Date("OxA-28797",5698,33)

{

Outlier(0.05);

z=0.7;

};

Curve("Marine13","Marine13.14c");

Delta_R("LocalMarine",123,28);

R_Date("OxA-28619",5692,30)

{

Outlier(0.05);

z=0.25;

};

Interval("Interval JE0087");

Span("Span JE0087");

};

Boundary("End 1");

};

};

### JE5641

Plot()

{

Outlier_Model("G",T(5),U(0,4),"t");

Curve("Marine13","Marine13.14c");

Delta_R("LocalMarine",188,44);

Sequence("Farasan")

{

Boundary();

Sequence("Sequence of JE5641")

{

Phase("Layer 3")

{

R_Date("OxA-30983",5922,39)

{

z=0.2;

Outlier(0.05);

};

R_Date("OxA-30984",5863,38)

{

z=0.2;

Outlier(0.05);

};

};

R_Date("OxA-30739",6015,40)

{

z=0.05;

Outlier(0.05);

};

Interval("Interval 5641");

Span("Span 5641");

};

Boundary();

};

};

### JE5642

Plot()

{

Outlier_Model("G",T(5),U(0,4),"t");

Curve("Marine13","Marine13.14c");

Delta_R("LocalMarine",188,44);

Sequence()

{

Boundary("Start 1");

Sequence("Sequence of JE5642")

{

R_Date("OxA-30869",5811,33)

{

z=0.6;

Outlier(0.05);

};

R_Date("OxA-31363",5701,35)

{

z=0.3;

Outlier(0.05);

};

R_Date("OxA-31165",5685,34)

{

z=0.05;

Outlier(0.05);

};

Interval("Interval 5642");

Span("Span 5642");

};

Boundary("End 1");

};

};

### JE5656

Plot()

{

Curve("Marine13","Marine13.14c");

Delta_R("LocalMarine",188,44);

Sequence("Farasan")

{

Boundary("start 5656");

Sequence("Sequence of JE5656")

{

R_Date("OxA-31454",5730,30)

{

z=0.4;

};

R_Date("OxA-31455",5741,37)

{

z=0.1;

};

Span("Span 5656");

Interval("Interval 5656");

};

Boundary("end 5656");

};

};

### JW1705

Plot()

{

Curve("Marine13","Marine13.14c");

Delta_R("LocalMarine",188,44);

Sequence("Farasan")

{

Boundary("start 1705");

Sequence("Sequence of JW1705")

{

Curve("Marine13","Marine13.14c");

Delta_R("LocalMarine",188,44);

R_Date("OxA-31167",6870,38)

{

z=1.00;

};

R_Date("OxA-31166",4842,32)

{

z=0.55;

};

R_Date("OxA-31168",3411,31)

{

z=0.1;

};

Interval("Interval 1705");

Span("Span 1705");

};

Boundary("End 1705");

};

};

### JW1727

Plot()

{

Outlier_Model("General",T(5),U(0,4),"t");

Curve("Marine13","Marine13.14c");

Delta_R("LocalMarine",123,28);

Sequence("JW1727")

{

Boundary("Start 1");

Sequence("JW1727")

{

Combine()

{

Curve("Marine13","Marine13.14c");

Delta_R("LocalMarine",123,28);

R_Date("OxA-28617",4701,28)

{

z=1.68;

Outlier("General", 0.05);

};

Curve("IntCal13","IntCal13.14c");

R_Date("OxA-27889",4287,29)

{

Outlier("General", 0.05);

z=1.68;

};

};

Curve("IntCal13","IntCal13.14c");

R_Date("OxA-27890",4202,29)

{

Outlier("General", 0.05);

z=0.95;

};

Curve("Marine13","Marine13.14c");

Delta_R("LocalMarine",123,28);

R_Date("OxA-34099", 4539, 33)

{

Outlier("General", 0.05);

z=0.50;

};Curve("Marine13","Marine13.14c");

Delta_R("LocalMarine",123,28);

R_Date("OxA-34098", 4759, 31)

{

Outlier("General", 0.05);

z=0.40;

};Curve("Marine13","Marine13.14c");

Delta_R("LocalMarine",123,28);

R_Date("OxA-28009",4851,31)

{

Outlier("General", 0.05);

z=0.15;

};

Interval("Interval of JW1727");

Span("Span of JW1727");

};

Boundary("End 1");

};

};

### JW1807

Plot()

{

Outlier_Model("General",T(5),U(0,4),"t");

Curve("Marine13","Marine13.14c");

Delta_R("LocalMarine",188,44);

Phase("JW1807")

{

Sequence("JW1807")

{

Boundary("Start JW1807");

Sequence("1")

{

Phase("Layer 25/26")

{

Curve("Marine13","Marine13.14c");

Delta_R("LocalMarine",188,44);

R_Date("28008", 5292, 30);

Curve("IntCal13","IntCal13.14c");

R_Date("28385", 4707, 30);

};

Boundary("25/17");

Curve("IntCal13","IntCal13.14c");

R_Date("28384", 4456, 31);

Curve("Marine13","Marine13.14c");

Delta_R("LocalMarine",188,44);

R_Date("28007", 5012, 30);

R_Date("28071", 4962, 29);

Interval("Interval of JW1807 ");

Span("Span of JW1807 ");

};

Boundary("End 1");

};

Sequence("JW1807")

{

Boundary("Start 1");

Sequence("JW1807")

{

Combine("Layer 25/26")

{

Curve("Marine13","Marine13.14c");

Delta_R("LocalMarine",188,44);

R_Date("OxA_28,008", 5292, 30)

{

Outlier("General", 0.05);

z=3.3;

};

Curve("IntCal13","IntCal13.14c");

R_Date("OxA_28,385", 4707, 30)

{

Outlier("General", 0.05);

z=3.3;

};

};

Curve("IntCal13","IntCal13.14c");

R_Date("OxA_28,384", 4456, 31)

{

Outlier("General", 0.05);

z=2.3;

};

Curve("Marine13","Marine13.14c");

Delta_R("LocalMarine",188,44);

R_Date("OxA_28,007", 5012, 30)

{

Outlier("General", 0.05);

z=0.7;

};

R_Date("OxA_28,071", 4962, 29)

{

Outlier("General", 0.05);

z=0.1;

};

Interval("Interval of JW1807");

Span("Span of JW1807");

};

Boundary("End 1");

};

};

};

### JW1864

Plot()

{

Outlier_Model("G",T(5),U(0,4),"t");

Curve("Marine13","Marine13.14c");

Delta_R("LocalMarine",188,44);

Sequence("JW1864")

{

Boundary("Start 1");

Sequence("Layer 6 to 1")

{

R_Date("OxA-31366", 5629, 34);

R_Date("OxA-31365", 5485, 34);

R_Date("OxA-31364", 5434, 34);

Interval("Interval 1864");

Span("Span 1864");

};

Boundary("End 1");

};

};

### JW2298

Plot()

{

Outlier_Model("General",T(5),U(0,4),"t");

Curve("Marine13","Marine13.14c");

Delta_R("LocalMarine",188,44);

Sequence("JW2298")

{

Boundary("Start 1");

Sequence("JW2298")

{

Phase("Layer 4")

{

R_Date("OxA-34102",5447,40)

{

z=1.85;

Outlier(0.05);

};

};

Boundary("Layer 4/3");

R_Date("OxA-34101",4920,35)

{

z=1.65;

Outlier(0.05);

};

R_Date("OxA-31368",5098,34)

{

z=1.5;

Outlier(0.05);

};

R_Date("OxA-34105",4973,31)

{

z=1.3;

Outlier(0.05);

};

R_Date("OxA-34107",5000,32)

{

z=1.2;

Outlier(0.05);

};

R_Date("OxA-34104",4981,33)

{

z=0.95;

Outlier(0.05);

};

R_Date("OxA-34100",5409,35)

{

z=0.6;

Outlier(0.05);

};

R_Date("OxA-34103",5150,32)

{

z=0.25;

Outlier(0.05);

};

R_Date("OxA-34106",4874,30)

{

z=0.2;

Outlier(0.05);

};

R_Date("OxA-31367",4846,32)

{

z=0.05;

Outlier(0.05);

};

Interval("JW2298");

Span("JW2298");

};

Boundary("End 1");

};

};

### JW3120

Plot()

{

Outlier_Model("G",T(5),U(0,4),"t");

Curve("Marine13","Marine13.14c");

Delta_R("LocalMarine",188,44);

Sequence()

{

Boundary("Start 1");

Sequence("Sequence of JW3120")

{

R_Date("OxA-28616",6208,31)

{

z=0.65;

Outlier(0.05);

};

R_Date("OxA-28697",5825,29)

{

z=0.1;

Outlier(0.05);

};

Interval("Interval 3120");

Span("Span 3120");

};

Boundary("End 1");

};

};

### JW5694

Plot()

{

Outlier_Model("G",T(5),U(0,4),"t");

Curve("Marine13","Marine13.14c");

Delta_R("LocalMarine",188,44);

Sequence("Farasan")

{

Boundary();

Sequence("Sequence of JW5694")

{

R_Date("OxA-30870",2902,29)

{

z=0.4;

Outlier(0.05);

};

R_Date("OxA-31170",2767,30)

{

z=0.3;

Outlier(0.05);

};

Interval("Interval 5694");

Span("Span 5694");

};

Boundary();

};

};

### JW5697

Plot()

{

Outlier_Model("G",T(5),U(0,4),"t");

Curve("Marine13","Marine13.14c");

Delta_R("LocalMarine",188,44);

Sequence("Farasan")

{

Boundary();

Sequence("Sequence of JW5697")

{

R_Date("OxA-31171",2220,27)

{

z=0.4;

Outlier(0.05);

};

R_Date("OxA-31487",2164,35)

{

z=0.1;

Outlier(0.05);

};

Interval("Interval JW5697");

Span("Span JW5697");

};

Boundary();

};

};

### JW5719

Plot()

{

Outlier_Model("G",T(5),U(0,4),"t");

Curve("Marine13","Marine13.14c");

Delta_R("LocalMarine",188,44);

Sequence("Farasan")

{

Boundary();

Sequence("Sequence of JW5719")

{

R_Date("OxA-31172",2500,29)

{

z=0.3;

Outlier(0.05);

};

R_Date("OxA-31173",2554,27)

{

z=0.1;

Outlier(0.05);

};

Interval("Interval of JW5719");

Span("Span of JW5719");

};

Boundary();

};

};
